# A Cascade of Conformational Switches in SARS-CoV-2 Frameshifting: Coregulation by Upstream and Downstream Elements

**DOI:** 10.1021/acs.biochem.4c00641

**Published:** 2025-02-05

**Authors:** Samuel Lee, Shuting Yan, Abhishek Dey, Alain Laederach, Tamar Schlick

**Affiliations:** † Department of Chemistry, New York University, New York, New York 10003, United States; ‡ Department of Biotechnology, National Institute of Pharmaceutical Education and Research-Raebareli (NIPER-R), Lucknow, Uttar Pradesh 226002, India; § Department of Biology, University of North Carolina at Chapel Hill, Chapel Hill, North Carolina 27599, United States; ∥ Courant Institute of Mathematical Sciences, New York University, New York, New York 10012, United States; ⊥ NYU-ECNU Center for Computational Chemistry, NYU Shanghai, Shanghai 200062, PR China; # NYU Simons Center for Computational Physical Chemistry, New York University, New York, New York 10003, United States

## Abstract

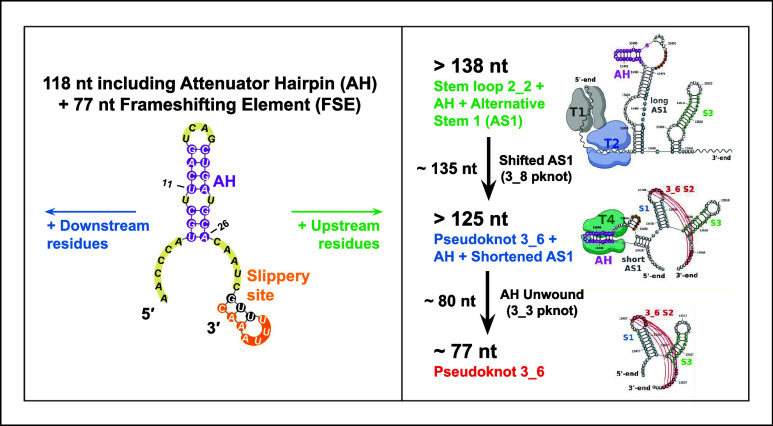

Targeting ribosomal frameshifting has emerged as a potential therapeutic intervention strategy against COVID-19. In this process, a −1 shift in the ribosomal reading frame encodes alternative viral proteins. Any interference with this process profoundly affects viral replication and propagation. For SARS-CoV-2, two RNA sites associated with ribosomal frameshifting are positioned on the 5′ and 3′ of the frameshifting residues. Although much attention has been focused on the 3′ frameshift element (FSE), the 5′ stem-loop (attenuator hairpin, AH) can play a role. Yet the relationship between the two regions is unknown. In addition, multiple folds of the FSE and FSE-containing RNA regions have been discovered. To gain more insight into these RNA folds in the larger sequence context that includes AH, we apply our graph-theory-based modeling tools to represent RNA secondary structures, “RAG” (RNA-As-Graphs), to generate conformational landscapes that suggest length-dependent conformational distributions. We show that the AH region can coexist as a stem-loop with main and alternative 3-stem pseudoknots of the FSE (dual graphs 3_6 and 3_3 in our notation) but that an alternative stem 1 (AS1) can disrupt the FSE pseudoknots and trigger other folds. A critical length for AS1 of 10-bp regulates key folding transitions. Together with designed mutants and available experimental data, we present a sequential view of length-dependent folds during frameshifting and suggest their mechanistic roles. These structural and mutational insights into both ends of the FSE advance our understanding of the SARS-CoV-2 frameshifting mechanism by suggesting how alternative folds play a role in frameshifting and defining potential therapeutic intervention techniques that target specific folds.

## Introduction

**Figure 1:**
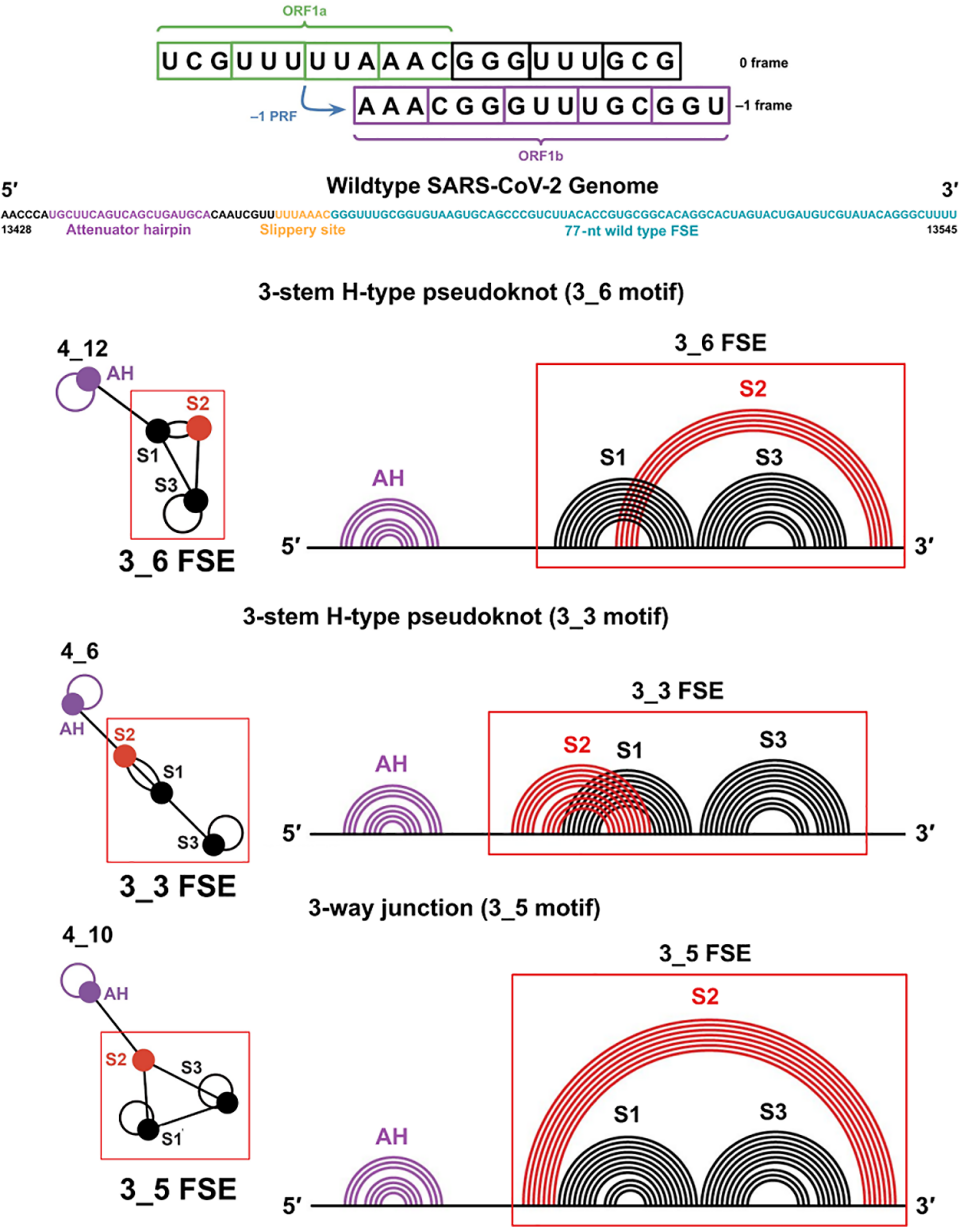
SARS-CoV-2 FSE sequence and three associated 2D motifs of 118-nt FSE-containing RNA folds emerging from this work. To illustrate the frameshifting process, the 0-frame and −1-frame codons are labeled with the overlapping regions of ORF1a and ORF1b marked. The attenuator hairpin, the 7-nt slippery site, and the downstream stimulatory pseudoknot are highlighted. For each 2D structure containing AH and FSE that adopts one of three possible folds, including the 3-stem H-type 3_6 pseudoknot, the alternative 3_3 pseudoknot, and the three-way junction 3_5 (unknotted RNA), corresponding dual graphs and arc plots are shown, with color-coded stems and loops labeled.

The COVID-19 pandemic has inspired innovative research into coronavirus proteins and viral RNAs, spanning many areas of protein dynamics, RNA structure and folding, and structural biophysics. Such research has been sustained because new therapeutic approaches are needed for recurrent viral threats. The frameshifting process has been a promising area of research with long-term therapeutic potential against viral infections.^1−6^ RNA frameshifting is regulated by the frameshifting element (FSE) of the RNA virus, located in the open reading frame ORF1a,b region of SARS-CoV-2’s viral genome that codes for the polyproteins necessary for viral protein synthesis (Figure 1). However, ORF1a and ORF1b overlap by a single nucleotide in coronavirus genomes: ORF1b starts from the −1 reading frame compared to ORF1a. The FSE is responsible for the programmed −1 ribosomal frameshift (−1 PRF), where translating ribosomes shift their reading frames by one nucleotide in the 5′ direction (−1). After this frameshift is complete, the ribosomes can decode the ORF1b polyproteins.^7^

The FSE of coronavirus has been characterized as a pseudoknot and associated with the −1 PRF slippage event at the 7-residue slippery site.^8−13^ The SARS-CoV-2 FSE is an 84-nt segment and contains, from 5′ to 3′, a 7-nt slippery site (UUUAAAC) and a 77-nt downstream RNA structure (see Figure 1).

A 20-residue attenuator hairpin (AH) upstream (3′-end) of the FSE may also be involved in frameshifting.^14,15^ The AH may downregulate the frameshifting process, causing ribosomes to dissociate from the mRNA before receiving the −1 PRF signal. Thus, the AH contributes to control a specific ratio of ORF1a to ORF1b polyprotein products, thereby helping optimize viral protein synthesis and replication.^16,17^

Although prior studies have characterized the 2D and 3D structures of the FSE, including flanking sequences, the exact folding interplay between the AH and FSE regions and the influence of these regions on frameshifting have yet to be fully elucidated.

Previous studies have underscored the significance of long-range RNA interactions in influencing the structure and function of the pseudoknot and the FSE within the SARS-CoV-2 RNA genome. For instance, Manfredonia et al.,^18^ Lan et al.,^19^ and Ziv et al.^20^ highlighted the importance of context-dependent effects on the FSE structure, emphasizing that local and global RNA folding can impact functional outcomes. Additionally, a study by Andrews et al.^21^ identified a segment within AS1 that exhibits statistically significant sequence covariation, providing strong evidence for its structural conservation and suggesting the incorporation of more upstream nucleotides. Building upon these insights, particularly regarding 2D and 3D RNA folding and the context-dependent effects on the FSE structure, would provide a valuable framework to interpret structural and functional results and integrate them into a broader understanding of SARS-CoV-2 RNA biology.

In our previous work, we explored the multiple-conformational landscape of the SARS-CoV-2 downstream FSE using our graph-theoretic approach, “RAG” (RNA-As-Graphs), which uses tree and dual graphs to represent, study, predict, and design RNA secondary (2D) structures.^22−26^ With our coarse-grained dual graphs, we delineated the alternative RNA structures of the downstream FSE and designed mutants via inverse folding^27^ that transform the FSE topology into others.^28,29^ Our FSE landscape includes the dominant 3-stem pseudoknot, which we term dual graph 3_6 (confirmed by X-ray, NMR, and Cryo-EM^2,30−34^), the alternative pseudoknot 3_3, and the three-way junction 3_5 (boxes in Figure 1).^29^

The three RNA motifs in Figure 1 contain the same Stems 1 and 3, but different Stem 2, which involves the 3′-end (in 3_6) or 5′-end (in 3_3). The 3_6 pseudoknot has also been confirmed by various methods,^2,30−33^ mostly for downstream sequences, but also with upstream sequences including the AH and a large portion of AS1.^34^

In our first FSE work,^28^ we designed four double mutants for 77-nt RNAs that transform the 3_6 pseudoknot into stem-loops 2_1 and 3_2, the three-way junction 3_5, and a 3_3 pseudoknot with S2 and S3 intertwined (rather than S1 and S2 in 3_3 in Figure 1). These 77-nt mutants were embedded in 114-nt RNAs called M1-M4 in ref.^35^ and experimentally tested by DMS-MaP. As expected, DMS-MaP data for 114-nt versions of our four 77-nt mutants^28^ yield 2_1, 3_2, and 3_5 for M1–M3 (see Results in ref.^35^ and Figure S1).

Later in ref.^29^, we also designed 77-nt minimal mutants (2 to 6 residues changed) to strengthen the 3_3, 3_6, and 3_5 FSE structures (termed M3_3, M3_6, and M3_5, respectively;^29^ see mutant sequences in Table 1 and also Figure 3 later). Pekarek et al.^35^ also reported that our earlier mutants decreased frameshifting by an order of magnitude (from 25.6% to 1–1.3%, including M3_5 mentioned here).^28^ More recent experiments show that our structure-stabilizing and transition-suppressing mutants reduce frameshifting by an order of magnitude,^36^ confirming our hypothesis that structural changes in FSE stem formation and overall folding play a significant role in fine-tuning the frameshifting process.

**Table 1:**
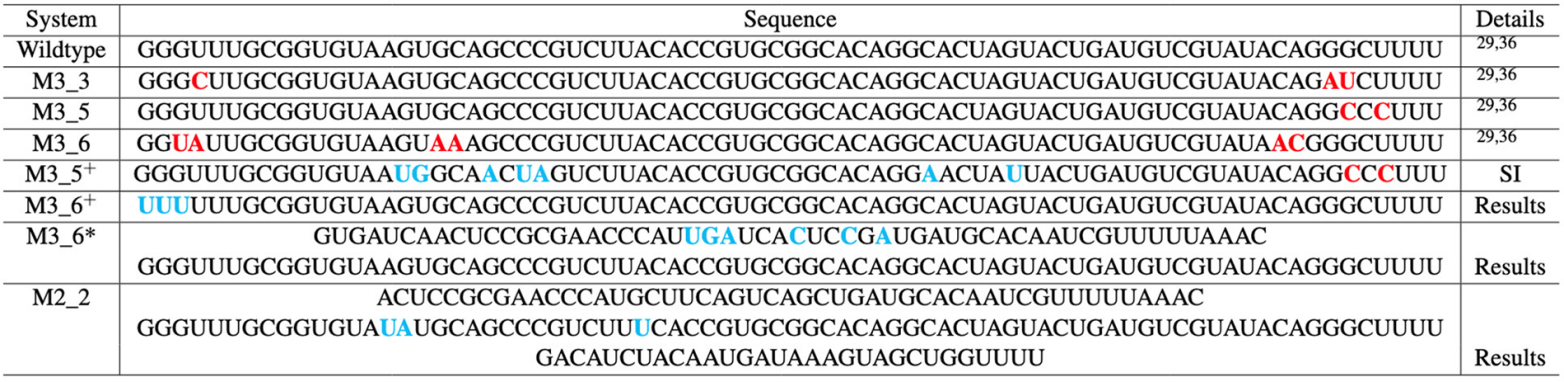
Sequences of the Central 77-nt FSE and Long FSE-Containing Constructs in the Mutants Studied in This Work^a^^,^^b^

a Mutations from ref.^29^ (M3_3, M3_6, and M3_5) are in bold and cyan.

b Mutations designed in this work (M3_5+, M3_6+, M3_6*, and M2_2) are in bold and cyan.

**Figure 2:**
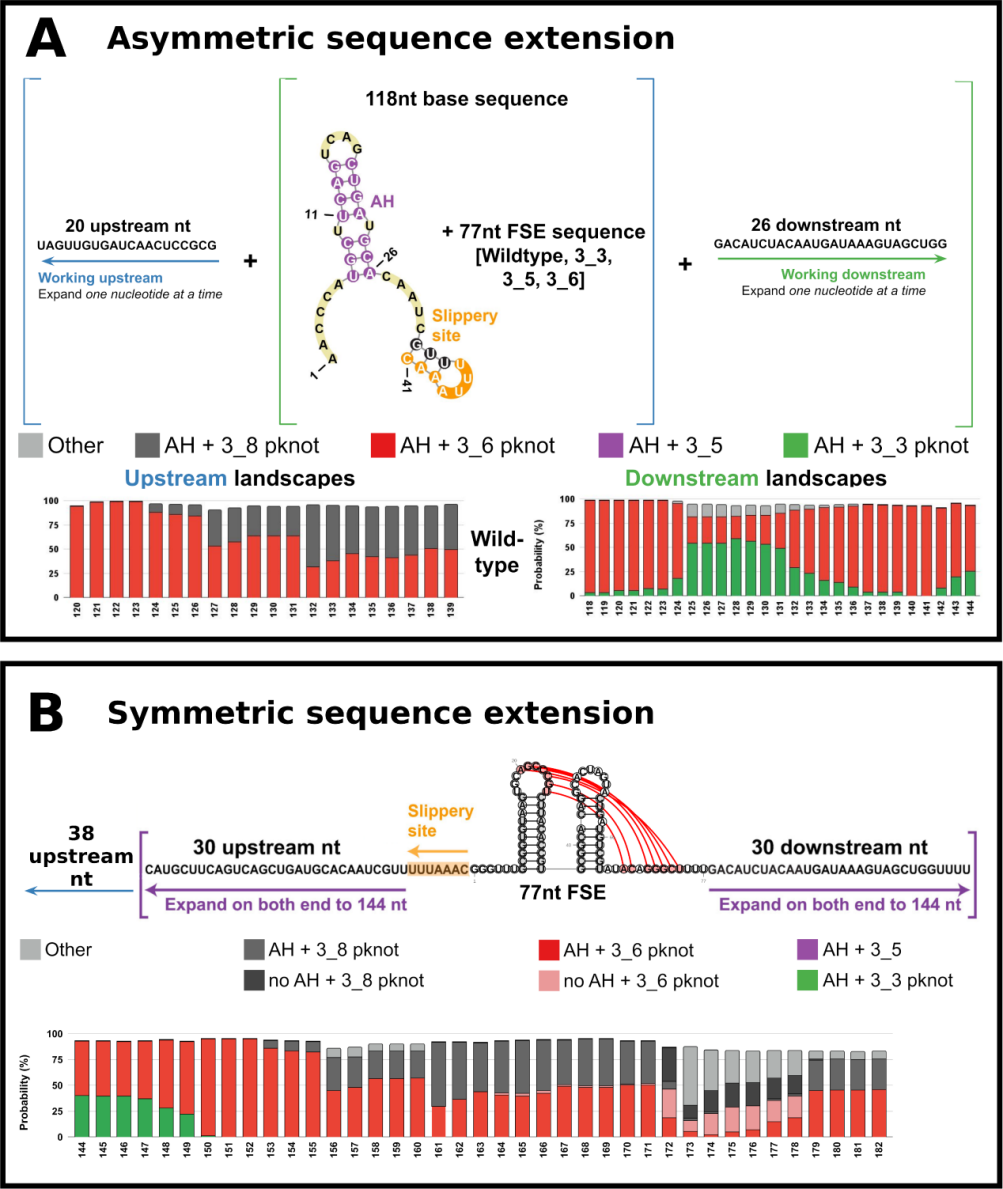
Conformational landscapes of the wild-type FSE with upstream or downstream extensions of sequence (top) and symmetric extensions of sequence (bottom). (A) In the conformational landscapes with increasing upstream or downstream sequence, the ensemble of 2D folds at each sequence length is predicted by NUPACK.^38^ For each length, probabilities of 2D folds are calculated from the Boltzmann factor, and all structures containing AH and 3_6, 3_3, or 3_5 motifs are individually summed. Motifs containing 3_6 and AH are in red; motifs containing 3_3 and AH are in green; and motifs containing 3_5 and AH are in purple. See Figure 4 for examples of folds at different lengths. (B) The bottom landscape is computed with a symmetric extension on both ends of the 144-nt segment containing the FSE.

Here, we continue to explore the length-dependent RNA folding via graph-theory-based modeling and chemical probing experiments to discover the relationship between AH formation and various alternative forms of the downstream stimulatory sequence and relate these folds to frameshifting. Specifically, we predict RNA folds as sequence increases via *conformational landscapes* (introduced in the studies^29,37^) as shown in Figure 2 and connect this information to experimental information for specific RNA lengths.

Nonpseudoknot structures such as stem-loops have been identified in our earlier RNA motifs^28,29^ and reported by others for long RNA lengths.^18,19,39−41^ These stem-loops can coexist with AH in long sequence constructs. We have proposed that structural transitions among these three, and likely other motifs, exist and play an important role in frameshifting.^37^ We continue to investigate this proposal in this study.

Specifically, we find here that the 3_3 and 3_6 FSE pseudoknots can coexist with AH in the wild-type sequence and that a switch between the two pseudoknots (3_3 and 3_6) depends on whether the residues upstream or downstream of the 77-nt FSE are exposed. Namely, in long sequence constructs, alternative stem AS1 blocks pseudoknot formation and favors simple stem-loops in the downstream FSE. By designing new mutants to block either AH formation or the pseudoknot, and considering new and published experimental data, we describe the structural relation between the upstream AH and downstream FSE. We further develop a mechanistic picture of ribosomal frameshifting involving multiple ribosomes and a dynamic folding/unfolding cascade during translation. Key in our mechanism are structural switches between unknotted stem-loops (when AS1 forms) and 3-stem pseudoknot/junction during the folding/refolding cycles. These folds and refolding transitions in a complex landscape define new avenues for targeting ribosomal frameshifting.

Our manuscript is organized by first introducing the wild-type landscapes and, second, discussing different FSE-containing RNA folds in light of experiments and other computations of wild-type and mutant systems. This combined information leads us to describe the frameshifting process as a cascade of conformational switches in the subsection that follows. The background material for graph representation, inverse folding, conformational landscapes, and DMS-MaPseq experiments is described in the “Materials and Methods” section.

## Materials and Methods

### RAG Dual Graphs and Inverse Folding

**Figure 3:**
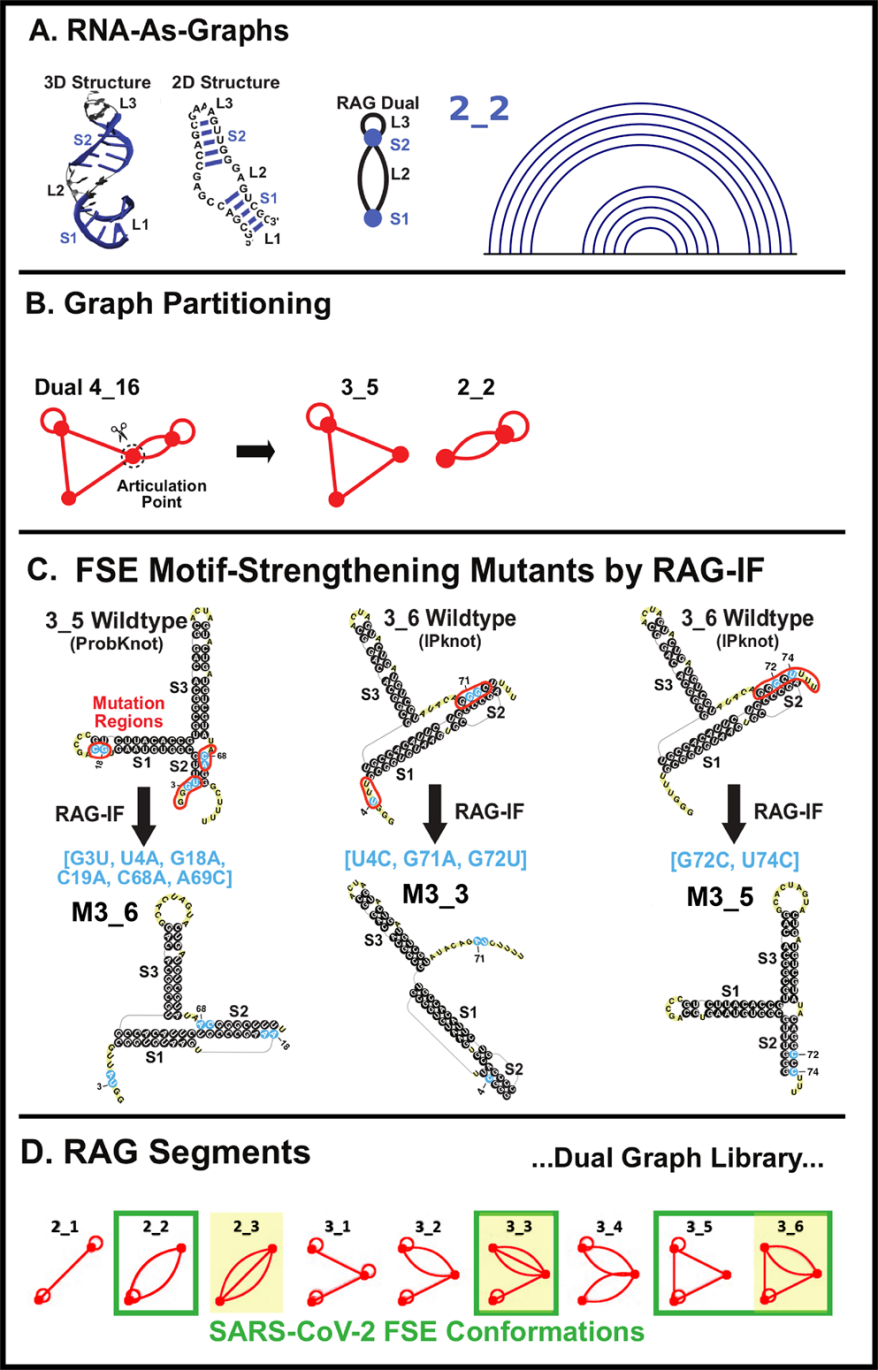
RNA modeling by graphs, atomic 2D and 3D models, and FSE applications: RAG elements and motif design. (A) Dual graph representation example of an RNA structure. Stems are denoted as vertices and single-stranded regions as edges. See ref. (22) for more details. (B) Graph partitioning, keeping pseudoknots and junctions intact, and subgraph representations. (C) Inverse folding design algorithm RAG-IF for obtaining minimal mutations that yield a target motif. See refs. (29) and (46) for details. (D) Dual graph library segments for available RNA structures, with SARS-CoV-2 FSE motifs highlighted. See ref.^47^ for details.

To represent RNA 2D structures in our RNA-As-Graphs (RAG) framework, double-stranded regions (stems) are denoted as vertices, and single-stranded regions (bulges, loops, and junctions) are edges in dual graphs.^22,42,43^ Hairpin loops are represented as self-edges, and unpaired residues at the 5′ and 3′ ends are ignored. See an introductory graph theory figure here (Figure 3), Figure 4 in ref.^25^, and a recent review in Comprehensive Computational Chemistry.^44^

**Figure 4:**
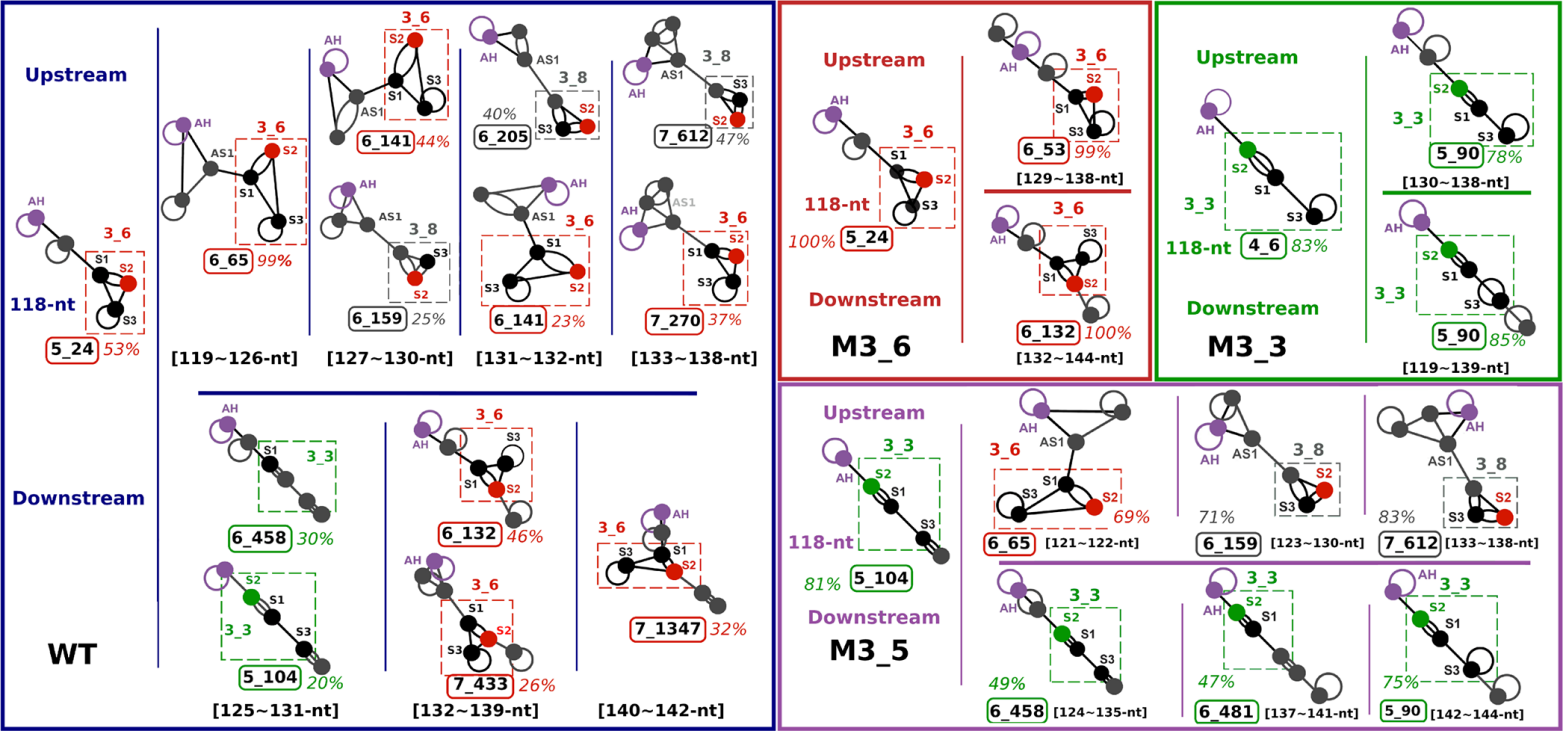
Dominant fold motifs for the FSE wild-type and mutant systems in this work with additional downstream and upstream sequences. Dual graphs of the dominant motif at certain sequence lengths are shown with AH labeled in purple and the central FSE motif in boxes. For each system, the dominant motif is shown for the 118-nt base sequence on the left. Dominant motifs from the landscapes with additional downstream sequences are on the top, and those from upstream landscapes are on the bottom. The FSE subgraphs are labeled in red for 3_6, green for 3_3, and gray for 3_8. Whole AH-FSE motifs are labeled with the highest percentage in the noted sequence range and boxed in colors according to the same grouping from the landscapes in Figure 2.

We use our inverse-folding protocol RAG-IF^27^ to mutate the FSE sequence to fold onto target dual-graph motifs. In brief, RAG-IF has three steps: 1) identify mutation regions and target 2D structures, 2) produce candidate sequences by mutations using a genetic algorithm (GA), and 3) optimize the mutated sequence pool by sorting and retaining only minimal or essential mutations that fold onto the target graph. GAs mimic evolution in nature, where the template sequence undergoes iterations of random mutation, crossover, and selection, and those with high fitness are retained. The fitness is determined by the Hamming distance. The nominated sequences are folded by IPknots^45^ and further examined by NUPACK.^38^ Sequences that achieve the target folding are further optimized to obtain the minimal mutations by removing the nonessential mutations.^27^

### Selection of Mutation Regions in Designed Mutants by RAG-IF

#### M3_6^+^

We select S1 5′-strand residues as the mutation region in order to break the G-C pairs that stabilize AS1 and restore S1.

#### M3_6*

AH residues are selected as the mutation region to break the base pairs and change the fold of the AH region. To test the independence of the folding of AH and AS1, AS1 residues are not selected to mutate.

#### M2_2

S1 residues are selected as the mutation region to disrupt S1 base pairs and support the 2_2 stem-loop.

#### M3_5^+^

We compare the wild-type FSE graph and the target graph to identify the smallest possible mutation regions required for the transformation. Considering that most of the motifs generated from appending downstream residues to the M3_5 mutant contain a 3_3 FSE, we focused on breaking the 3_3 pseudoknot to allow the 3_5 FSE to form in its place. We specifically selected the downstream residues of S2 or S3 as the mutation site. Here, we describe how this is accomplished for our eight most successful target graphs: 6_458, 5_104, 5_90, 6_481, 7_2207, 6_390, 4_6, and 7_2365. We focus on the motif topology and allow the lengths of the stems and loops to vary.

### Conformational Landscape Calculation

For each variant (wild type, PSM M3_3, M3_5, M3_6), we start with the base 41-nt sequence: AACCCAUGCUUCAGUCAGCUGAUGCACAAUCGUU- UUUAAAC.

The base 41-nt sequence contains the AH and the slippery site. The sequence is selected according to Pekarek et al.’s^35^ FSE-V2 construct. We append the 77-nt unique sequence corresponding to each variant (see Table 1) after the slippery site (UUUAAAC, see underlined above), and see the full sequence of 118-nt in Table S1.

Working downstream, we add the 26-nt sequence GACAUCUACAAUGAUAAAGUAGCUGG one nucleotide at a time, appending it to the 3′-end of each template sequence (base 41-nt + unique 77-nt). Working upstream, we add the 20-nt sequence UAGUUGUGAUCAACUCCGCG one nucleotide at a time, appending it to the 5′-end of each template sequence.

For each coronavirus FSE, we use NUPACK v3.2.2^38,48^*subopt* mode and option *-pseudo* to predict RNA secondary structure ensembles for lengths from 118 to 138-nt adding upstream sequence and lengths from 118 to 144-nt adding downstream sequence. NUPACK is used after comparing the prediction performances of different RNA prediction software packages (shown in Table S2 and Figure S5). The output contains the predicted 2D structures, together with their free energy estimates. We then use the Boltzmann distribution to compute the partition function *Z* and the probability *p*_*i*_ for each 2D structure *i*.
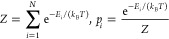
1where *E*_*i*_ is the free energy estimate of structure *i*, *k*_B_ is the Boltzmann constant, and *T* is the room temperature (37 °C).

For each length, we apply our dual graph representation and sum the probabilities of structures that correspond to the same graph. Only graphs with probabilities ≥1% are retained, and representative 2D structures are recorded for these graphs.

Next, we identify the minimal motifs within these example structures that involve the 118-nt FSE-containing regions. Once we identify all minimal motifs, we sum up the probabilities of dual graphs that correspond to the same minimal motif and retain only motifs with probabilities ≥5%.

### DMS-MaPseq Chemical Probing

#### In Vitro RNA Chemical Probing Read by Mutational Profiling

The 156-nt SARS-CoV-2 FSE-containing construct was synthesized as G-blocks from Integrated DNA Technologies (IDT). The whole construct was flanked at both the 5′ and 3′ ends by RNA cassettes.^49^ For *in vitro* transcription of the 156-nt FSE-containing construct, a T7 promoter region was added to its 5′-end. Transcription was performed using a T7 HiScribe RNA synthesis kit (New England Biolabs). The generated RNA was subjected to DNase treatment (TURBODNase), which was further purified using a PureLink RNA Mini Kit (Invitrogen) and quantified using a NanoDrop. The standardized cassettes used are specifically designed to form hairpin structures upstream and downstream of the construct, which have been shown to allow more accurate structure predictions based on the DMS data as the amplicon primers do not cover the signal for the 5′ and 3′ ends of the construct.

For chemical probing, 6 μg of purified synthetic RNA was denatured at 65 °C for 5 min and snap-cooled in ice. Following denaturation, folding buffer (100 mM KCl, 10 mM MgCl_2_, 100 mM Bicine, pH 8.3) was added to the denatured RNA, and the whole reaction mixture was incubated at 37 °C for 10 min. The folded RNA was further treated with 10 μL of 1:10 ethanol-diluted dimethyl sulfate (DMS). For control, an equivalent volume of ethanol was added to the folded RNA. Probing was initiated at 37 °C for 5 min and was quenched afterward using 100 μL of 20% β-mercaptoethanol (β-ME). Modified and unmodified RNAs were purified using the PureLink RNA Mini Kit (Invitrogen) and quantified using a NanoDrop.

#### Library Construction, Sequencing, and Data Processing

Both chemically modified and unmodified RNAs were reverse transcribed using a gene-specific primer (Table 2) complementary to the 3′ RNA cassette and Superscript II reverse transcriptase under error-prone conditions as previously described.^50^ The cDNA generated was further purified using a G50 column (GE Healthcare) and subjected to second strand synthesis (NEBNext Second Strand Synthesis Module). For constructing next-generation sequencing libraries, the double-stranded (ds) cDNA was PCR amplified using primers directed against 5′ and 3′ RNA cassettes and the NEB Q5 HotStart polymerase (NEB). To introduce unique barcodes, a secondary PCR was performed using TruSeq primers (NEB).^50^ The resultant libraries were purified using Ampure XP (Beckman Coulter) beads and quantified using a Qubit dsDNA HS Assay kit (Thermo Fisher). For quality checks, libraries were subjected to an Agilent Bioanalyzer 2100. Final libraries were sequenced as 2 × 151 paired-end reads on the Illumina MiSeq platform. To calculate mutation frequency in both chemically modified (DMS-treated) and control (ethanol-treated) RNA samples, the ShapeMapper2 algorithm was used.^51^ Chemical modifications on each RNA nucleotide were calculated using the following equation:
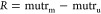
2where *R* is the chemical reactivity, mutr_m_ is the mutation rate calculated for chemically modified RNA, and mutr_u_ is the mutation rate calculated for unmodified control RNA samples.^50^

**Table 2:** Primers Used for PCR and Reverse Transcription of 156-nt SARS-CoV-2 FSE-Containing Sequence

Primer	Sequence
3′ Cassette-RT	GAACCGGACCGAAGCCCG
5′ Cassette-Fwd	CCCTACACGACGCTCTTCCGATCTNNNNNCCCAGGGTCTG
3′ Cassette-Rev	GACTGGAGTTCAGACGTGTGCTCTTCCGATCTNNNNNCCCAGGGGCTG

#### In Vitro RNA Chemical Probing and Mutational Profiling

Different length constructs of SARS-CoV-2 FSE (77-nt, 87-nt, 156-nt, and 222-nt) based on our earlier simulation studies and 5NIA-based mutational profiling^29^ were synthesized as G-blocks from Integrated DNA Technologies (IDT). These were chosen to probe the influence of additional nucleotide sequences and lengths on the overall conformation landscape of the SARS-CoV-2 FSE. Our 87-nt construct contains a slippery sequence, while the 77-nt construct is devoid of it. All constructs were flanked at the 5′ and 3′ ends by RNA cassettes^49^ with a T7 promoter region added to the 5′-end of each construct for in vitro transcription of RNA using T7 RNA polymerase from the T7 HiScribe RNA synthesis kit (New England Biolabs). The synthesized RNA was DNase treated (TURBODNase) and purified using a PureLink RNA Mini Kit (Invitrogen) and quantified using a NanoDrop.

Chemical probing of each construct was performed as described earlier.^36^ Briefly, for chemical probing, 6 μg of purified in vitro transcribed RNA was denatured at 65 °C for 5 min and snap-cooled in ice. Following denaturation, folding buffer (100 mM KCl, 10 mM MgCl_2_, 100 mM Bicine, pH 8.3) was added, and the whole reaction was incubated at 37 °C for 10 min. The reaction was further treated with 10 μL of 1:10 ethanol-diluted dimethyl sulfate (DMS). For control, an equivalent volume of ethanol was added to the folded RNA. Probing was initiated by incubating the reaction mixture at 37 °C for 5 min followed by quenching with an additional 100 μL of 20% β-mercaptoethanol (β-ME). Modified and unmodified RNAs were purified using the PureLink RNA Mini Kit and quantified using a NanoDrop.

### RNA Structure Predictions with DMS-MaPseq Data

We only analyze the 87-nt regions of longer constructs for SHAPEKNOTS, DREEM, DRACO, and DANCE-MaP as done earlier.^36^

#### DREEM

Using the “detection of RNA folding ensembles using expectation-maximization” (DREEM) algorithm,^52^ alternative structures were directly identified from the sequencing reads from DMS-MaPseq.

Using an expectation–maximization technique, DREEM clusters the reads into discrete groups based on patterns of DMS-induced mutations. Log-likelihood is maximized to obtain the DMS modification rate per base for each cluster. In this work, a maximum of *K* = 3 clusters was used to group the bit-vectors. The resulting DMS reactivities for each cluster were then used as constraints for ShapeKnots^53^ predictions. Hence, distinct structural clusters with their relative ratios result in different folds, which represents the heterogeneity of the RNA secondary structure.

#### DRACO

We also applied the DRACO algorithm,^54^ which performs deconvolution of alternative RNA conformations from mutational profiling experiments with a combination of spectral clustering and fuzzy clustering, to validate the structure prediction. Spectral clustering is performed for the sliding windows along the transcript, allowing the optimal number of coexisting conformations (clusters) to be automatically identified from the eigengaps. Following the determination of the number of clusters, fuzzy clustering is carried out to allow bases to be weighted according to their affinity for each cluster. DRACO then reconstructs the overall mutational profiles by merging overlapping windows with the same number of clusters. DRACO reports consecutive sets of windows with varying amounts of clusters separately.

The pair-end reads were merged by PEAR^55^ and mapped to the reference sequence using the rf-map tool^56^ (parameters: -b2 -cqo -ctn -mp “–very-sensitive-local”). The resulting BAM files were then analyzed with the rf-count tool to produce MM files (-r -m -mm -na -ni). MM files were analyzed with DRACO^54^ (parameters: −allNonInformativeToOne–nonInformativeToSurround–minClusterFraction 0.1), and deconvoluted mutation profiles were extracted from the resulting JSON files. Normalized reactivity profiles were obtained by first calculating the raw reactivity scores via the scheme by Zubradt et al.^57^ as the per-base ratio of the mutation count and the read coverage at each position, and then by 90% Winsorizing as a normalization method, using the rf-norm tool^56^ (parameters: -sm 4 -nm 2 -rb AC -mm 1). Data-driven RNA structure prediction was performed using ShapeKnots^53^ and the normalized reactivity profiles.

## Results

### Overview

In the subsequent sections, we generate conformational landscapes of frameshifting element-containing RNA sequences computationally (see note below) to analyze the various conformations of the SARS-CoV-2 systems as a function of increasing sequence length, mimicking ribosomal translation as introduced in the study.^37^

We begin with a sequence that contains AH and 3_6 FSE (also modeled by Pekarek et al.^35^). Adding *upstream* (5′) residues mimics the RNA sequence that the ribosome encounters when the genome is unwound, as the ribosome moves along the RNA transcript. For comparison, we also constructed landscapes with varying *downstream* (3′) sequence lengths to mimic the refolding when the ribosome moves further downstream. For added perspective, we also consider *symmetric* increases of sequence length, as in our previous study.^29^ Although our computed landscapes only suggest general trends, our prior experimental confirmation with SHAPE^29^ and current comparison with DMS-MaPseq chemical probing data lend confidence in this approach.

Starting from the 118-nt base sequence, which contains the upstream AH and downstream FSE, we use graph IDs to describe the RNA fold motifs in the conformational landscape succinctly, e.g., 20% pseudoknot 3_3 and 80% pseudoknot 3_6. Because sequences are longer than 77-nt, higher-order folds result (i.e., with more than 3 vertices), but these large graphs may contain the 3_6, 3_3, and 3_5 as subgraphs, and we color them accordingly (see Figure 1). The symmetric landscapes reflect both upstream and downstream extensions.

Our landscapes are generated by NUPACK^38^ (see the “Materials and Methods” section), which has yielded consistent results for our work and in comparison to experimental references before.^29^ NUPACK generates suboptimal structures that allow us to determine the conformational distribution (see information in the Supporting Information on the performance of other packages) representing the conformational thermodynamic equilibrium. The kinetic rate of transition processes among various motifs at different lengths is beyond the scope of the current study; note that our separate work explored the dynamic transition pathways between two SARS-CoV-2 FSE pseudoknots.^58^

In addition to our computational landscapes, our paper presents and analyzes new DMS-MaPseq FSE chemical probing data (see the “Materials and Methods” section) for the 156-nt FSE-containing sequence and designs four new mutants that strengthen 3_6, stem-loop 2_2, and three-way junction 3_5, all for long sequences. Previously published data are also analyzed with the mechanistic information gleaned from our landscapes.

### Wild-Type SARS-CoV-2 Conformational Landscapes

Figure 2 presents the landscapes for the wild-type system. At each RNA length, we fold the RNA “in silico” and report the percentages of each “fold” (denoted by its RAG graph ID) within the conformational ensemble. The color codes in our landscapes represent our 4-fold classes. Graphs that include AH with 3_6, 3_3, or 3_5 as subgraphs (see Figure 4), or other folds, are colored in different colors (red, green, purple, or gray). For example, starting at 118-nt, we have 95.2% of the 3_6 pseudoknot and 3.6% of the 3_3 pseudoknot (Figure 2A). Thus, the majority of the histogram bar is red (3_6 family), and the rest is green (for the 3_3 family) for this RNA length (see Figure 2A). The gray represents other folds, mostly stem-loops and large motifs containing them; stem-loops were a notable minority in our earlier work.^28^ Thus, for 118-nt, we obtain the 3_6 pseudoknot with AH (Figures 2A and 4), in full agreement with the recent cryo-EM structure of the Moss group.^34^

**Figure 5:**
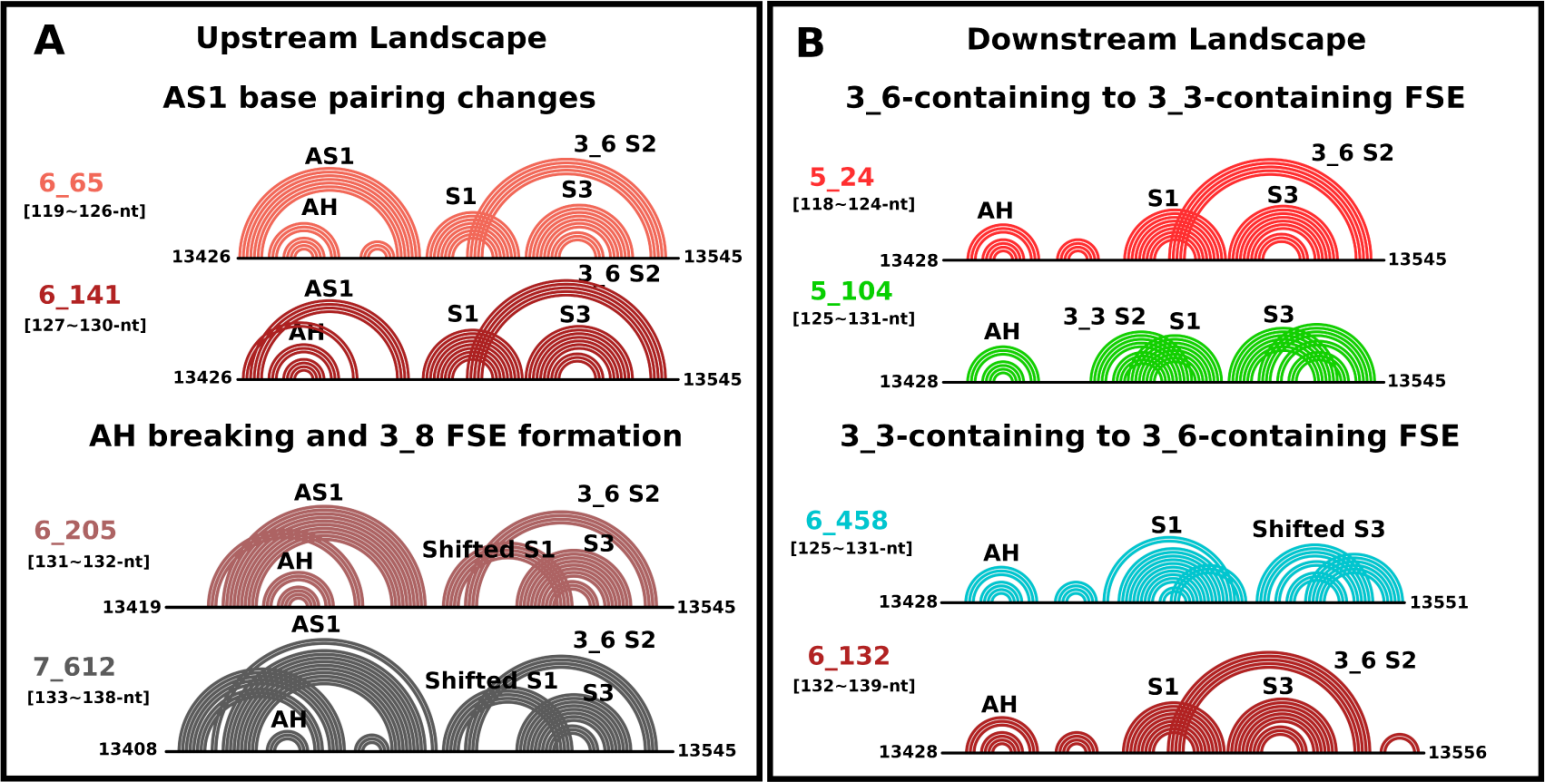
Transitions of 2D folding from the dominant motifs of FSE wild-type in this work with additional upstream (A) and downstream (B) sequences are shown in arc plots. 3_6 FSE-containing motifs are colored in red; similarly, 3_3 FSE-containing motifs are colored in green, and 3_8-containing motifs are colored in gray.

As we add downstream residues to the RNA, the FSE contains a stable AH with a downstream 3_6 pseudoknot until 125-nt; then, the majority adopts a 3_3 FSE pseudoknot instead. The exact dual graphs obtained at different lengths are shown in Figure 4. Figure 5 illustrates the shift from the AH and 3_6-containing fold to the AH and 3_3-containing fold as red to green stems, where S2 reforms at longer sequences. When sequences reach lengths above 132-nt, FSE motifs (dual graphs in Figure 4) combine AH and a stable 3_6 FSE, consistent with reported experimental results (Figure S2A).

As we add upstream residues to the wild-type FSE, an alternative stem 1 (AS1) upstream forms, protecting the AH and blocking the formation of 3_3 S2 and 3_5 S2 (Figure 5, right). This favors 3_6 topologies as well as a 3_8 pseudoknot (dual graphs in Figure 4 and related 2D folds in Figure 5, left). A minority of stem-loops (under Other) also results. The 3_8 pseudoknot can form from 3_6 S2 and S3 with a shifted S1. As clearly seen in Figure 5, right, AS1’s increasing size can block or alter S1.

To investigate the folding of the AH-FSE region more thoroughly, especially AS1 formation at long sequences, we also extend the 144-nt symmetric construct we previously developed in Figure 2B, which includes 30-nt upstream and downstream of the central FSE region. In this scenario of a longer upstream sequence, the 3_6 pseudoknot competes with 3_8, and both motifs coexist with AH. Additional base pairs strengthen AS1, blocking S1 and supporting 3_8 (see Figure S2B). The length 156-nt marks the emergence of a 12-bp AS1.

For this length, we capture a minority of stem-loops (under Other), with 3_6 and 3_8 folds. Experimental data support stem-loops for the 156-nt construct as a majority, but various analysis programs differ in their outcomes (Figure S2B). The DREEM algorithm^52^ reveals alternative conformations from DMS-MaPseq reads, showing the dominant stem-loop 2_2 without S1 and a minor alternative stem-loop with S1, 2_1. The differences noted from various deconvolution methods used to handle sequencing reads emphasize the prediction sensitivity to DMS reactivity profiles.

Together with our landscapes that reveal a growing portion of stem-loops for long lengths, we conclude that all these folds play a role and are possible in the FSE landscape (see also Discussion in Dey et al.^36^). Namely, our landscapes and DMS probing data all agree that this region of the SARS-CoV-2 genome adopts multiple competing conformations that are part of the conformational landscape and whose relative proportions are highly sensitive to the RNA length and to the analysis programs. Our landscape makes clear that 3_6 S2 can coexist with AH and that an AS1 longer than 10 bp results in shifted S1. Without S1, stem-loops (2_1 or 2_2) observed in multiple experimental studies^19,59,60^ and in long constructs by our DMS-MaPseq (156-nt, Figure S2B, and 222-nt FSE-containing sequence, Figure S6) are favored instead of the pseudoknot 3_6. DMS-MaPseq reactivity data are plotted in Figure S7.

### SARS-CoV-2 Conformational Landscapes for Pseudoknot Mutants

**Figure 6:**
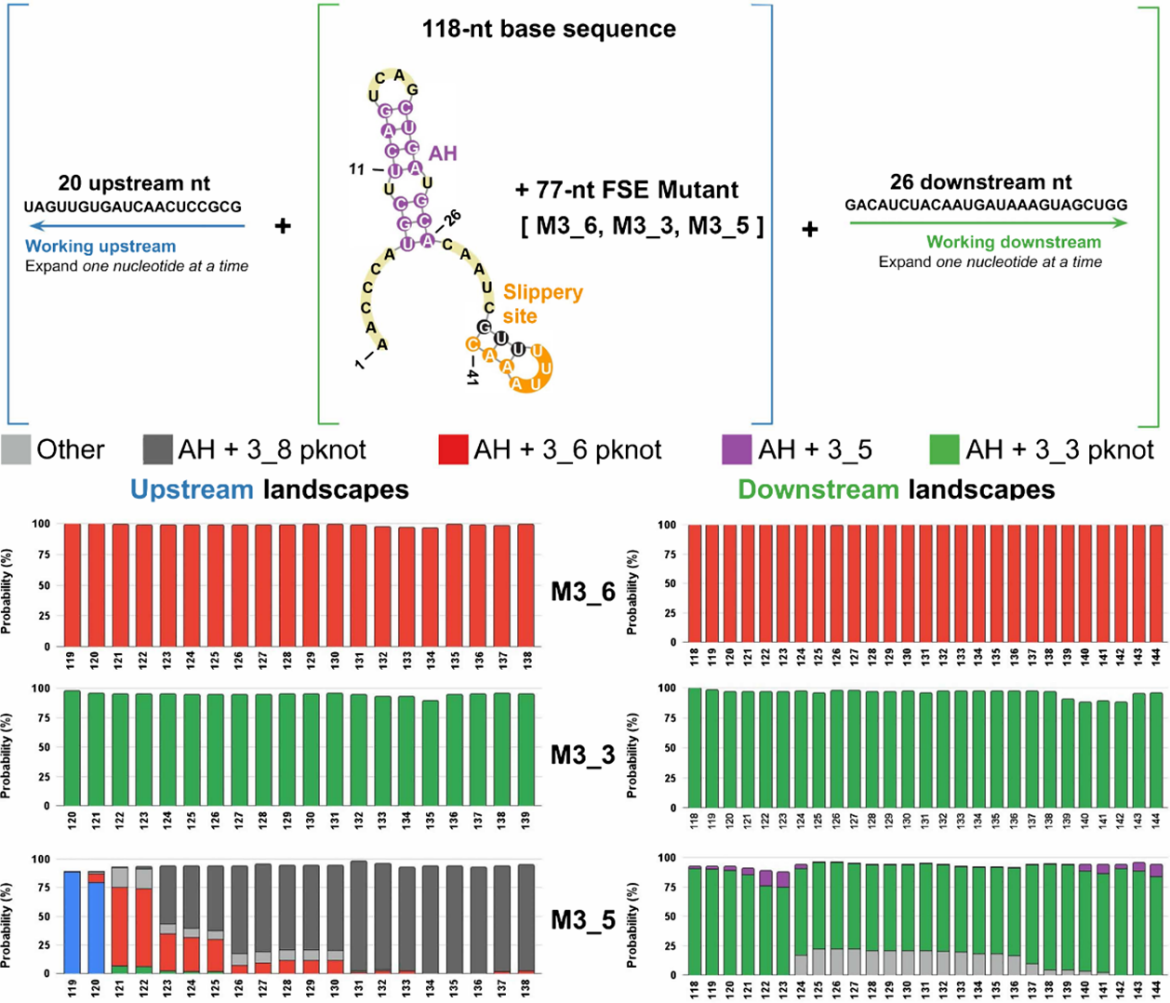
Conformational landscapes of our three motif strengthening mutants M3_6, M3_3, and M3_5 with expanding sequences upstream and downstream. Motifs containing 3_6 and AH are in red; motifs containing 3_3 and AH are in green; and motifs containing 3_5 and AH are in purple. See Figure 4 for examples of folds at different lengths.

In our previous work,^29^ we designed a series of pseudoknot-strengthening mutants to fold onto the desired FSE conformation at 77-nt. In Dey et al.,^36^ these mutants for 77-nt were shown to nearly abolish frameshifting, including M3_6 and M3_3 to stabilize these pseudoknots. To test how mutants that stabilized these conformational folds in short FSE-containing RNAs behaved in a longer sequence context, we explored the conformational landscapes for these mutants containing sequences in long constructs. When AH is considered, our M3_6 folds almost perfectly into a stable AH and a downstream 3_6 FSE (Figure 6), both when adding downstream or upstream residues. Additional hairpins are appended downstream or upstream of the AH-FSE region (Figure S8A). M3_3 also fares well in folding into both an upstream AH and a downstream 3_3 FSE when adding both downstream and upstream residues (for example, 5_90 in Figure 4). Adding upstream residues generates a stable AH along with a stable downstream 3_3 FSE. However, S3 can be replaced by downstream hairpins, e.g., 6_390, after appending downstream residues to M3_3 at 140–142-nt (shown in Figure S8B).

The stability of M3_5 for the three-way junction is examined via landscapes in Supporting Information. The design of a stable 3_5 (3-way junction) fold at a long sequence is given in Figure S9 based on the blocking of alternative dominant structures observed in the conformational landscapes.

### Conformational Switches in Frameshifting Folding Landscape

**Figure 7:**
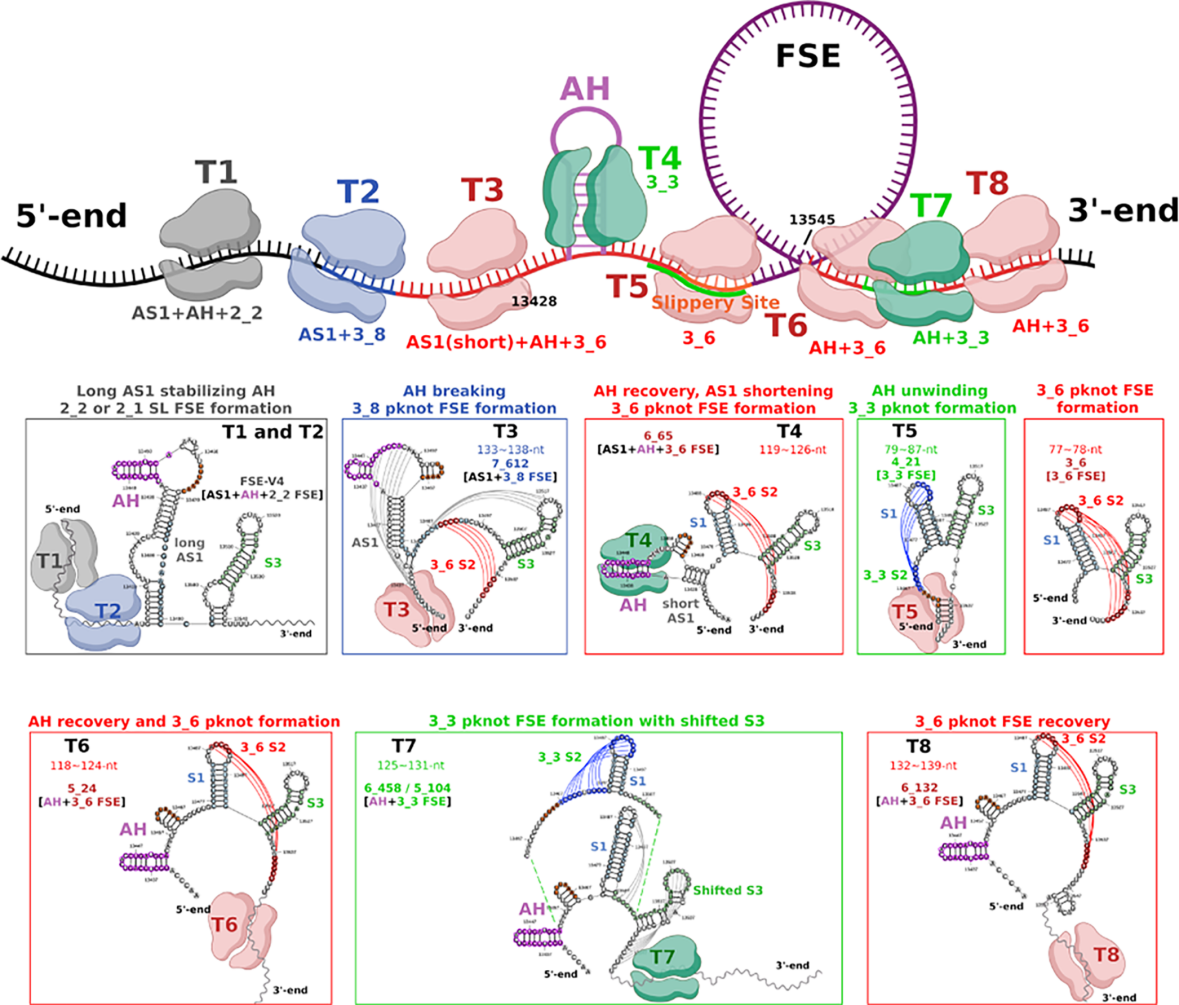
Proposed FSE conformational transitions resulting from unwinding by ribosomes during translation. In our top schematic diagram, the topology of the central FSE region changes as the ribosome moves along the RNA genome. Ribosomes located at timesteps T1 to T8 are colored based on the associated FSE fold motif: red for 3_6 pseudoknot, green for 3_3 pseudoknot, and gray for unknotted stem-loop 2_2. The RNA templates are boxed and colored to match the ribosome positions. The arrows indicate the range of ribosome unwinding of the local RNA. Folds of the AH-FSE region are shown in secondary structure visualization via RNAcanvas.^61^

Combining our conformational landscape information and experimental findings, we propose in Figure 7 that the length-dependent FSE folding follows this trajectory as the ribosome unwinds during translation. Namely, as the ribosome moves along the wild-type sequence (upstream sequence decreasing), our landscapes in Figure 2 reveal the following folding mechanisms of the AH-FSE template region:

(1) Long sequences promote the formation of unknotted stem-loops that contain an elongated AS1 of 12 base pairs or more and either a 2_2 (simple stem-loop) or 2_1 FSE **(template 1)**.
(2) Disruption of partial AS1 results in a shifted S1 in the predicted 3_8 motif (for central FSE) **(template 2)**.
(3) Further unwinding leads to a shortened AS1, AH, and 3_6 FSE **(template 3)**.
(4) Unwinding of AH results in 3_3 FSE formation when the slippery site is available for pairing **(template 4)**.
(5) When the ribosome’s movement renders the slippery site inaccessible, the 3_6 FSE is reinstated **(template 5)**.
(6) Additional downstream residues from the 77-nt central FSE support a 3_3 FSE (see 5_109, for example) **(template 6 to 7)**.
(7) As the ribosome further moves away from FSE, 3_6 FSE is reestablished, alongside AH **(template 8)**.

In general, short stems, for example, 3_3 S2 and 3_8 shifted S1, can form temporarily after adding sequences upstream or downstream, while long stems, such as 2_2 stems and S3, are more stable. This likely explains the stem-loop structures in many genome-wide structural analyses.^19,59^ In a separate work, we simulate the 3_3 to 3_6 pseudoknot transition, confirming its importance on aligning the FSE region in the narrow mRNA channel.^58^

In the remaining subsections, we explore mutant designs to confirm our sequence of RNA folds related to frameshifting. See Table 1.

### M3_6+ and M3_6*: AS1 or AH Blocking Mutation Design for 3_6 at Long Sequences to Stabilize Pseudoknot

**Figure 8:**
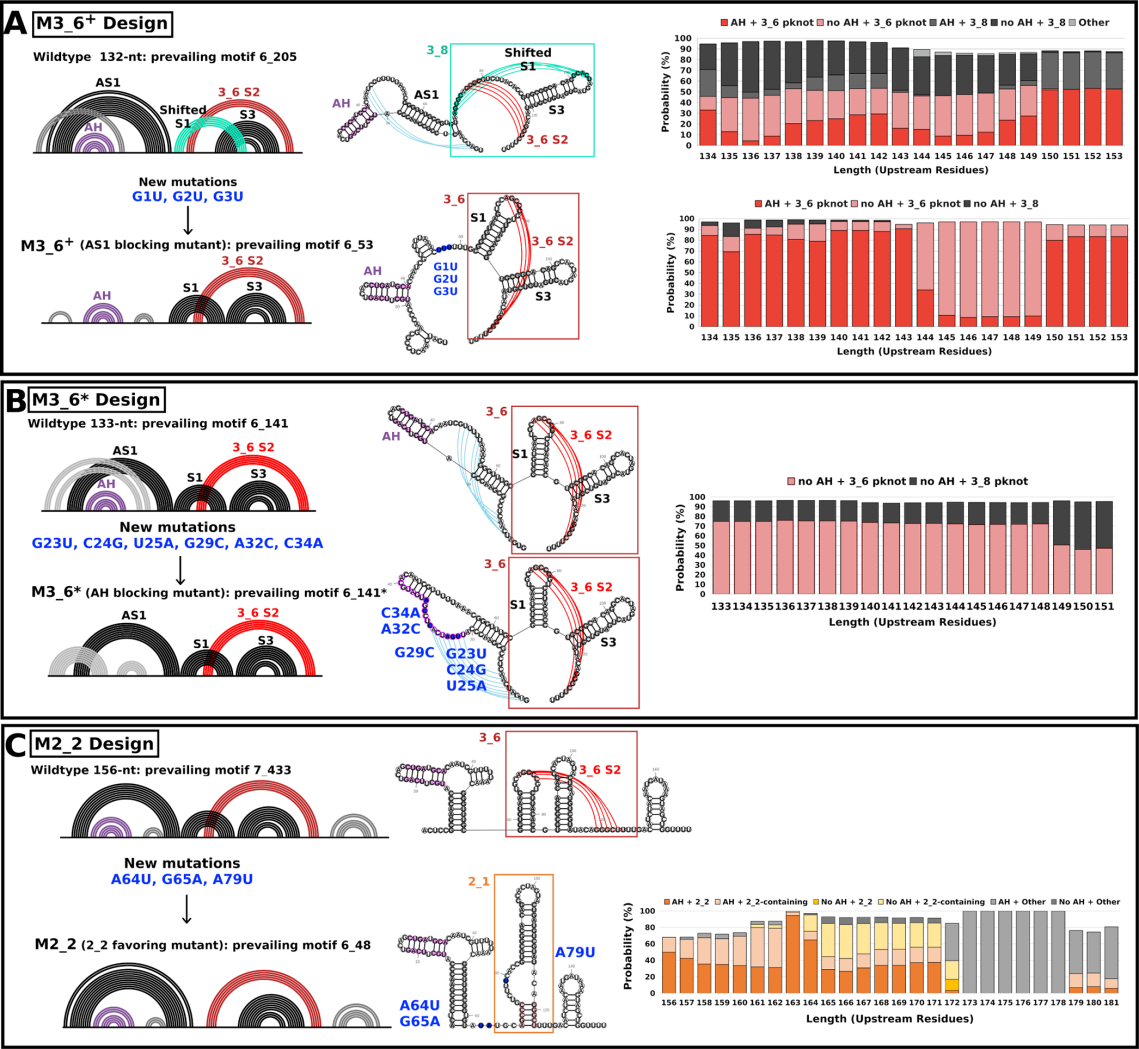
Mutant designs to block AS1 (M3_6^+^), block AH (mutant M3_6*), or favor stem-loop 2_2 by blocking S1 (M2_2) in long FSE-containing sequences. (A) To block AS1 and form the 3_6 pseudoknot, we manually mutate G1 to G3 in the 77-nt FSE region to U (denoted in blue) and examine the folding for 132-nt and stability in long RNAs (expanding upstream from 134 to 153-nt). Three G-C pairs in the 12 bp AS1 at 132-nt are disrupted, and thus, AS1 with 3_8 is blocked in our resulting M3_6^+^ mutant. (B) To block AH and form the 3_6 pseudoknot, we use RAG-IF and 2D structure prediction programs IPknot and NUPACK to screen and determine the minimal mutations. For the 133-nt FSE-containing sequence, we mutate the AH region and replace AH with a stem knotted with AS1 and a small hairpin. The conformational landscape expanding upstream shows the stability of the mutant motif. (C) To block S1 and favor the 2_2 stem-loop, we design the sequence with minimal mutations for the 156-nt FSE-containing sequence. Three mutations are introduced to destroy three base pairs (2 AU pairs and 1 GC pair) in S1 and thus favor the 2_2 stem-loop. The conformational landscape expanding upstream confirms our target stem-loop stability.

In our cascade of conformations and folds, AS1 length plays a critical role because a long AS1 (≥12-bp) blocks the formation of the 3_6 pseudoknot. To further validate our mechanistic landscape (Figure 7), we mutate the FSE region to block the formation of AS1. In Figure 8A, we examine the folding of the mutated sequence for 132-nt, where a 12-bp AS1 exists for the wild type. The G-U mutations indeed block AS1 formation and recover S1 in the 3_6 pseudoknot. We expand the sequence with additional upstream residues and see that the 3_6 FSE replaces folds with both AS1 and 3_8 and thereby dominates the landscape with high stability, as designed. We call this mutant M3_6^+^.

Alternatively, we stabilize the 3_6 pseudoknot by destroying AH through mutations in the hairpin region while maintaining AS1 and the 3_6 pseudoknot (M3_6*). From Figure 8B, we split AH strands into two stems, including an 8-bp stem knotted with AS1 and a 5-bp hairpin. Thus, the upstream stem-loop seen in our 156 and 222-nt FSE-containing sequences in Figures S2 and S6 and by others^19,35^ switches to a 3_6 pseudoknot.

### M2_2: Stem-Loop Favoring Mutation Design for Stem-Loop at Long Sequences

To strengthen stem-loop 2_2, we block the formation of S1 by mutating the FSE S1 region. S1 is inhibited by three mutations in our M2_2 mutant shown in Figure 8C. AS1 extends from 8-bp to 12-bp, which is consistent with the observations from our landscape. This automatic mutation design via RAG-IF confirms that disrupting S1 results in a 12-bp AS1 and 2_2 FSE, supporting our findings of the effect of the upstream sequence on the FSE pseudoknot and stem-loop conversion.

Details of a fourth mutant, M3_5+, to stabilize a three-way junction instead of the pseudoknot 3_3, are described in Section SIV and Figure S9.

Combining the findings from this study with those of earlier research,^28,29^ the robust picture as shown in Figure 7 emerges of how AH and AS1 compete/interfere with the short sequence FSE pseudoknot 3_6.

## Discussion

Programmed ribosomal frameshifting (PRF) is thought to rely on pseudoknots or stem-loop RNA folds that are critical to pausing. However, many PRF systems are associated with multiple folds of the frameshifting element. For SARS-CoV-2, the picture is especially confusing since several stem-loops and pseudoknots have been detected experimentally and by computation. What, then, is the role of these alternative folds? In this work, based on computations and DMS-MaPseq experiments, we have suggested that a kinetic sequence of events of folding and refolding directs PRF. Understanding this complex dance presents an opportunity to develop therapeutic intervention strategies, such as mutations by RNA-guided gene editing tools, to interfere with this process. FSE-ribosome interactions are also important to consider as FSE folding is constrained by the ribosome in the cellular context. For example, we showed in our work focusing on the 3_3 to 3_6 pseudoknot transition that this transition is triggered by tension formed in the RNA strand due to ribosomal clashes and that the transition releases this tension and places the RNA properly into the narrow ribosomal channel.^58^

Our proposal in Figure 7 elucidates the length and context-dependent folding of the AH-FSE region and its impact on frameshifting, stemming from conformational landscapes for different folding/refolding scenarios. When AS1 does not form, AH is compatible at low sequence lengths with the 3_6 and 3_3 pseudoknots. As AS1 develops at medium lengths, it replaces S1 and leads to a shifted S1 in 3_8 with a conserved AH. With additional upstream and downstream sequences, the anchoring helix AS1 expands and blocks FSE pseudoknot formation. When the ribosome unwinds the long anchoring helix AS1, the downstream sequence can refold back into the 3_6 pseudoknot.

To reinforce our mechanistic understanding of the evolving template-driven folding and unfolding events that occur in the SARS-CoV-2 FSE, we have designed four mutants that favor specific motifs while destroying certain stems or hairpins. Our M3_6^+^ mutant blocks AS1 altogether and favors 3_6. The M3_6^*^ mutant disrupts AH base pairs and results in a 3_6 pseudoknot containing AS1. The M2_2 mutant blocks S1 and supports a stem-loop FSE in combination with a 12-bp AS1. The mutant M3_5^+^ restores 3_5 and blocks 3_3 for long sequences. These four mutants favor 3_6, 2_2, or 3_5 while destroying AS1, AH, or S1, respectively. These design experiments support our proposed cascade of FSE-containing folds in Figure 7 and may be useful for antiviral approaches targeting a specific conformer. Their effect on frameshifting can be further examined by experiments.

Because each virus has evolved to create optimal rates of −1 PRF by balancing more and less stable structural features,^17^ alternative structures play an important role in frameshifting and viral propagation. The AH has been shown to trigger ribosome dissociation from the mRNA and thus decrease the fraction of ribosomes available to frameshifting.^17^ The AH can form without AS1, perhaps explaining why the hairpin remains when the sequence is less conserved in the upstream region. While its formation does not affect the downstream FSE region, AS1 involving the spacer and S1 sequences has a large impact on downstream FSE. This is consistent with the observation that the spacer sequence plays a significant role in frameshifting efficiency.^2,62^

The formation of these structures depends on translation rates between conformers and ribosomal pausing.^63^ Forming a stable and unwinding-resistant conformation to impede translating ribosomes may be valuable for developing efficient frameshifting elements,^63^ as in AH protected by a stable AS1.

Taking into account the limitations of various computational pipelines in producing alternative clusters, predicting pseudoknots, and integrating SHAPE/DMS chemical probing data, our conformational landscapes, as presented here, together with experimental data, yield a robust picture, as shown in Figure 7.

The conclusions regarding the multiple competing conformations observed in the FSE landscape are supported by the integration of both the DMS experimental data and computational results. While the DMS data indicate a population distribution that differs from the computational predictions, this discrepancy highlights the inherent flexibility and heterogeneity of the FSE region. The computational models provide a snapshot of energetically favorable conformations, while the experimental DMS data reflect a dynamic ensemble of structures present in solution. Together, these findings suggest that the FSE does not adopt a single static fold but rather exists as part of a conformational landscape, where multiple structures coexist and compete. The relative proportions of these structures are influenced by factors such as RNA length and experimental conditions, as noted. This dynamic folding behavior aligns with the proposed mechanisms, where structural plasticity is crucial for frame-shift regulation, allowing the RNA to respond sensitively to its sequence context and cellular environment. By demonstrating the coexistence of multiple conformations, our study underscores the importance of both computational predictions and experimental validation in capturing the full complexity of the RNA folding landscape.

Indeed, our computational work and experimental validations^29,35^ suggest an interactive model of AH-FSE structure modulated by translating ribosomes during frameshifting and largely influenced by AS1 formation. Blocking AS1 in our M3_6^+^ mutant may also allow for more complex folds to form (Figure 8B). Additional experimental and computational perspectives, especially in the context of the ribosome, will undoubtedly shed further insight into this complex and important process.

## Conclusions

Our study elucidates the intricate interplay between upstream and downstream elements that regulate ribosomal frameshifting in SARS-CoV-2. By leveraging graph-theory-based modeling and experimental validation, we have demonstrated that the attenuator hairpin (AH) and frame-shifting element (FSE) engage in a dynamic cascade of conformational transitions influenced by sequence length and structural competition. These transitions, characterized by the coexistence and mutual exclusivity of pseudoknots (3_6 and 3_3) and stem-loops, underscore the regulatory complexity of programmed −1 ribosomal frameshifting (PRF).

Our findings reveal that AS1 formation is a critical determinant in modulating the structural landscape of the FSE, favoring either pseudoknot formation or alternative folds, as shown in Figure 7. Importantly, our designed mutants (Table 1 and Figure 8) validate the mechanistic framework by stabilizing specific conformers and disrupting others. These results highlight potential therapeutic avenues, including targeting specific RNA conformations to hinder frameshifting and thereby impede viral replication.

The broader implications of this study extend beyond SARS-CoV-2, providing a framework to investigate RNA structural plasticity and its functional roles in other viral systems. Future investigations integrating ribosome-bound structures and kinetic modeling will refine our understanding of RNA folding landscapes under physiological conditions, offering deeper insights into the relationship between RNA structure and function in viral translation and replication.

## Data Availability

Raw fastq.gz files for the DMS-MaPseq experiments can be downloaded from SRA project accession PRJNA1086021. The input and output files from DREEM and DRACO pipelines are deposited in Zenodo DOI 10.5281/zenodo.14518803, which contains reactivity profiles and structure predictions for 87-nt, 156-nt, and 222-nt FSE-containing sequences.

## Supporting Information Available

Additional experimental details and materials, including methods for DMS chemical probing and computational analysis, are provided. Figures S1–S10 provide visualizations of RNA folding predictions, chemical probing data, motif stability, and mutant designs. Tables S1–S3 detail mutant sequences, software predictions, and motif fractions (PDF)

## References

[ref1] Sun Y.; Abriola L.; Niederer R. O.; Pedersen S. F.; Alfajaro M. M.; Monteiro V. S.; Wilen C. B.; Ho Y.-C.; Gilbert W. V.; Surovtseva Y. V.; et al. Restriction of SARS-CoV-2 replication by targeting programmed −1 ribosomal frameshifting. Proc. Natl. Acad. Sci. U.S.A. 2021, 118, e2023051118 10.1073/pnas.2023051118. 34185680 PMC8256030

[ref2] Bhatt P. R.; Scaiola A.; Loughran G.; Leibundgut M.; Kratzel A.; Meurs R.; Dreos R.; O’Connor K. M.; McMillan A.; Bode J. W.; Thiel V.; Gatfield D.; Atkins J. F.; Ban N. Structural basis of ribosomal frameshifting during translation of the SARS-CoV-2 RNA genome. Science 2021, 372, 1306–1313. 10.1126/science.abf3546. 34029205 PMC8168617

[ref3] Varricchio C.; Mathez G.; Pillonel T.; Bertelli C.; Kaiser L.; Tapparel C.; Brancale A.; Cagno V. Geneticin shows selective antiviral activity against SARS-CoV-2 by interfering with programmed −1 ribosomal frameshifting. Antiviral Res. 2022, 208, 105452 10.1016/j.antiviral.2022.105452. 36341734 PMC9617636

[ref4] Munshi S.; Neupane K.; Ileperuma S. M.; Halma M. T. J.; Kelly J. A.; Halpern C. F.; Dinman J. D.; Loerch S.; Woodside M. T. Identifying Inhibitors of −1 Programmed Ribosomal Frameshifting in a Broad Spectrum of Coronaviruses. Viruses 2022, 14 (2), 177 10.3390/v14020177. 35215770 PMC8876150

[ref5] Yang M.; Olatunji F. P.; Rhodes C.; Balaratnam S.; Dunne-Dombrink K.; Seshadri S.; Liang X.; Jones C. P.; Le Grice S. F. J.; Ferré-D’Amaré A. R.; Schneekloth J. S. J. Discovery of Small Molecules Targeting the Frameshifting Element RNA in SARS-CoV-2 Viral Genome. ACS Med. Chem. Lett. 2023, 14, 757–765. 10.1021/acsmedchemlett.3c00051. 37312842 PMC10258829

[ref6] Huang S.-H.; Chen S.-C.; Wu T.-Y.; Chen C.-Y.; Yu C.-H. Programmable modulation of ribosomal frameshifting by mRNA targeting CRISPR-Cas12a system. iScience 2023, 26, 108492 10.1016/j.isci.2023.108492. 38125012 PMC10730746

[ref7] Kelly J. A.; Woodside M. T.; Dinman J. D. Programmed −1 Ribosomal Frameshifting in coronaviruses: A therapeutic target. Virology 2021, 554, 75–82. 10.1016/j.virol.2020.12.010. 33387787 PMC7833279

[ref8] Brierley I.; Digard P.; Inglis S. C. Characterization of an efficient coronavirus ribosomal frameshifting signal: Requirement for an RNA pseudoknot. Cell 1989, 57, 537–547. 10.1016/0092-8674(89)90124-4. 2720781 PMC7133225

[ref9] Napthine S.; Liphardt J.; Bloys A.; Routledge S.; Brierley I. The role of RNA pseudoknot stem 1 length in the promotion of efficient −1 ribosomal frameshifting 1. J. Mol. Biol. 1999, 288, 305–320. 10.1006/jmbi.1999.2688. 10329144 PMC7126229

[ref10] Kontos H.; Napthine S.; Brierley I. Ribosomal Pausing at a Frameshifter RNA Pseudoknot Is Sensitive to Reading Phase but Shows Little Correlation with Frameshift Efficiency. Mol. Cell. Biol. 2001, 21, 8657–8670. 10.1128/MCB.21.24.8657-8670.2001. 11713298 PMC100026

[ref11] Baranov P. V.; Henderson C. M.; Anderson C. B.; Gesteland R. F.; Atkins J. F.; Howard M. T. Programmed ribosomal frameshifting in decoding the SARS-CoV genome. Virology 2005, 332, 498–510. 10.1016/j.virol.2004.11.038. 15680415 PMC7111862

[ref12] Plant E. P.; Pérez-Alvarado G. C.; Jacobs J. L.; Mukhopadhyay B.; Hennig M.; Dinman J. D. A Three-Stemmed mRNA Pseudoknot in the SARS Coronavirus Frameshift Signal. PloS Biol. 2005, 3, e172 10.1371/journal.pbio.0030172. 15884978 PMC1110908

[ref13] Namy O.; Moran S. J.; Stuart D. I.; Gilbert R. J. C.; Brierley I. A mechanical explanation of RNA pseudoknot function in programmed ribosomal frameshifting. Nature 2006, 441, 244–247. 10.1038/nature04735. 16688178 PMC7094908

[ref14] Cho C.-P.; Lin S.-C.; Chou M.-Y.; Hsu H.-T.; Chang K.-Y. Regulation of Programmed Ribosomal Frameshifting by Co-Translational Refolding RNA Hairpins. PLoS One 2013, 8, e62283 10.1371/journal.pone.0062283. 23638024 PMC3639245

[ref15] Hu H.-T.; Cho C.-P.; Lin Y.-H.; Chang K.-Y. A general strategy to inhibiting viral −1 frameshifting based on upstream attenuation duplex formation. Nucleic Acids Res. 2016, 44, 256–266. 10.1093/nar/gkv1307. 26612863 PMC4705660

[ref16] Su M.-C.; Chang C.-T.; Chu C.-H.; Tsai C.-H.; Chang K.-Y. An atypical RNA pseudoknot stimulator and an upstream attenuation signal for −1 ribosomal frameshifting of SARS coronavirus. Nucleic Acids Res. 2005, 33, 4265–4275. 10.1093/nar/gki731. 16055920 PMC1182165

[ref17] Plant E. P.; Rakauskaitė R.; Taylor D. R.; Dinman J. D. Achieving a Golden Mean: Mechanisms by Which Coronaviruses Ensure Synthesis of the Correct Stoichiometric Ratios of Viral Proteins. J. Virol. 2010, 84, 4330–4340. 10.1128/JVI.02480-09. 20164235 PMC2863758

[ref18] Manfredonia I.; Nithin C.; Ponce-Salvatierra A.; Ghosh P.; Wirecki T. K.; Marinus T.; Ogando N. S.; Snijder E. J.; van Hemert M. J.; Bujnicki J. M.; Incarnato D. Genome-wide mapping of SARS-CoV-2 RNA structures identifies therapeutically-relevant elements. Nucleic Acids Res. 2020, 48, 12436–12452. 10.1093/nar/gkaa1053. 33166999 PMC7736786

[ref19] Lan T. C. T.; Allan M. F.; Malsick L. E.; Woo J. Z.; Zhu C.; Zhang F.; Khandwala S.; Nyeo S. S. Y.; Sun Y.; Guo J. U.; et al. Secondary structural ensembles of the SARS-CoV-2 RNA genome in infected cells. Nat. Commun. 2022, 13, 1128 10.1038/s41467-022-28603-2. 35236847 PMC8891300

[ref20] Ziv O.; Price J.; Shalamova L.; Kamenova T.; Goodfellow I.; Weber F.; Miska E. A. The Short- and Long-Range RNA-RNA Interactome of SARS-CoV-2. Mol. Cell 2020, 80, 1067–1077.e5. 10.1016/j.molcel.2020.11.004. 33259809 PMC7643667

[ref21] Andrews R. J.; O’Leary C. A.; Tompkins V. S.; Peterson J. M.; Haniff H.; Williams C.; Disney M. D.; Moss W. N. A map of the SARS-CoV-2 RNA structurome. Nar: Genomics Bioinf. 2021, 3, lqab043 10.1093/nargab/lqab043. PMC8140738 34046592

[ref22] Gan H. H.; Pasquali S.; Schlick T. Exploring the repertoire of RNA secondary motifs using graph theory; implications for RNA design. Nucleic Acids Res. 2003, 31, 2926–2943. 10.1093/nar/gkg365. 12771219 PMC156709

[ref23] Zahran M.; Sevim Bayrak C.; Elmetwaly S.; Schlick T. RAG-3D: A search tool for RNA 3D substructures. Nucleic Acids Res. 2015, 43, 9474–9488. 10.1093/nar/gkv823. 26304547 PMC4627073

[ref24] Baba N.; Elmetwaly S.; Kim N.; Schlick T. Predicting Large RNA-Like Topologies by a Knowledge-Based Clustering Approach. J. Mol. Biol. 2016, 428, 811–821. 10.1016/j.jmb.2015.10.009. 26478223 PMC4789128

[ref25] Schlick T. Adventures with RNA graphs. Methods 2018, 143, 16–33. 10.1016/j.ymeth.2018.03.009. 29621619 PMC6051918

[ref26] Meng G.; Tariq M.; Jain S.; Elmetwaly S.; Schlick T. RAG-Web: RNA structure prediction/design using RNA-As-Graphs. Bioinformatics 2020, 36, 647–648. 10.1093/bioinformatics/btz611. 31373604 PMC7999136

[ref27] Jain S.; Tao Y.; Schlick T. Inverse folding with RNA-As-Graphs produces a large pool of candidate sequences with target topologies. J. Struct. Biol. 2020, 209, 107438 10.1016/j.jsb.2019.107438. 31874236 PMC7042079

[ref28] Schlick T.; Zhu Q.; Jain S.; Yan S. Structure-Altering Mutations of the SARS-CoV-2 Frameshifting RNA Element. Biophys. J. 2021, 120, 1040–1053. 10.1016/j.bpj.2020.10.012. 33096082 PMC7575535

[ref29] Schlick T.; Zhu Q.; Dey A.; Jain S.; Yan S.; Laederach A. To Knot or Not to Knot: Multiple Conformations of the SARS-CoV-2 Frameshifting RNA Element. J. Am. Chem. Soc. 2021, 143, 11404–11422. 10.1021/jacs.1c03003. 34283611 PMC8315264

[ref30] Jones C. P.; Ferré-D’Amaré A. R. Crystal structure of the severe acute respiratory syndrome coronavirus 2 (SARS-CoV-2) frameshifting pseudoknot. RNA 2022, 28, 239–249. 10.1261/rna.078825.121. 34845084 PMC8906546

[ref31] Roman C.; Lewicka A.; Koirala D.; Li N.; Piccirilli J. The SARS-CoV-2 Programmed – 1 Ribosomal Frameshifting Element Crystal Structure Solved to 2.09 Å Using Chaperone-Aßisted RNA Crystallography. ACS Chem. Biol. 2021, 16, 1469–1481. 10.1021/acschembio.1c00324. 34328734 PMC8353986

[ref32] Wacker A.; Weigand J. E.; Akabayov S. R.; Altincekic N.; Bains J. K.; Banijamali E.; Binas O.; Castillo-Martinez J.; Cetiner E.; Ceylan B.; Chiu L.-Y.; Davila-Calderon J.; Dhamotharan K.; Duchardt-Ferner E.; Ferner J.; Frydman L.; Fürtig B.; Gallego J.; Grün J. T.; Hacker C.; Haddad C.; Hähnke M.; Hengesbach M.; Hiller F.; Hohmann K. F.; Hymon D.; de Jesus V.; Jonker H.; Keller H.; Knezic B.; Landgraf T.; Löhr F.; Luo L.; Mertinkus K. R.; Muhs C.; Novakovic M.; Oxenfarth A.; Palomino-Schätzlein M.; Petzold K.; Peter S. A.; Pyper D. J.; Qureshi N. S.; Riad M.; Richter C.; Saxena K.; Schamber T.; Scherf T.; Schlagnitweit J.; Schlundt A.; Schnieders R.; Schwalbe H.; Simba-Lahuasi A.; Sreeramulu S.; Stirnal E.; Sudakov A.; Tants J.-N.; Tolbert B. S.; Vögele J.; Weiß L.; Wirmer-Bartoschek J.; Wirtz Martin M. A.; Wöhnert J.; Zetzsche H. Secondary structure determination of conserved SARS-CoV-2 RNA elements by NMR spectroscopy. Nucleic Acids Res. 2020, 48, 12415–12435. 10.1093/nar/gkaa1013. 33167030 PMC7736788

[ref33] Zhang K.; Zheludev I. N.; Hagey R. J.; Haslecker R.; Hou Y. J.; Kretsch R.; Pintilie G. D.; Rangan R.; Kladwang W.; Li S.; Wu M. T.-P.; Pham E. A.; Bernardin-Souibgui C.; Baric R. S.; Sheahan T. P.; D’Souza V.; Glenn J. S.; Chiu W.; Das R. Cryo-EM and antisense targeting of the 28-kDa frameshift stimulation element from the SARS-CoV-2 RNA genome. Nat. Struct. Mol. Biol. 2021, 28, 747–754. 10.1038/s41594-021-00653-y. 34426697 PMC8848339

[ref34] Peterson J. M.; Becker S. T.; O’Leary C. A.; Juneja P.; Yang Y.; Moss W. N. Structure of the SARS-CoV-2 Frameshift Stimulatory Element with an Upstream Multibranch Loop. Biochemistry 2024, 63, 1287–1296. 10.1021/acs.biochem.3c00716. 38727003 PMC12345388

[ref35] Pekarek L.; Zimmer M. M.; Gribling-Burrer A.-S.; Buck S.; Smyth R.; Caliskan N. Cis-mediated interactions of the SARS-CoV-2 frameshift RNA alter its conformations and affect function. Nucleic Acids Res. 2023, 51, 728–743. 10.1093/nar/gkac1184. 36537211 PMC9881162

[ref36] Dey A.; Yan S.; Schlick T.; Laederach A. Abolished frameshifting for predicted structure-stabilizing SARS-CoV-2 mutants: Implications to alternative conformations and their statistical structural analyses. RNA 2024, 30, 1437–1450. 10.1261/rna.080035.124. 39084880 PMC11482603

[ref37] Yan S.; Zhu Q.; Jain S.; Schlick T. Length-dependent motions of SARS-CoV-2 frameshifting RNA pseudoknot and alternative conformations suggest avenues for frameshifting suppreßion. Nat. Commun. 2022, 13, 4284 10.1038/s41467-022-31353-w. 35879278 PMC9310368

[ref38] Zadeh J. N.; Steenberg C. D.; Bois J. S.; Wolfe B. R.; Pierce M. B.; Khan A. R.; Dirks R. M.; Pierce N. A. NUPACK: Analysis and design of nucleic acid systems. J. Comput. Chem. 2011, 32, 170–173. 10.1002/jcc.21596. 20645303

[ref39] Iserman C.; Roden C. A.; Boerneke M. A.; Sealfon R. S. G.; McLaughlin G. A.; Jungreis I.; Fritch E. J.; Hou Y. J.; Ekena J.; Weidmann C. A.; et al. Genomic RNA Elements Drive Phase Separation of the SARS-CoV-2 Nucleocapsid. Mol. Cell 2020, 80, 1078–1091.e6. 10.1016/j.molcel.2020.11.041. 33290746 PMC7691212

[ref40] Sanders W.; Fritch E. J.; Madden E. A.; Graham R. L.; Vincent H. A.; Heise M. T.; Baric R. S.; Moorman N. J. Comparative analysis of coronavirus genomic RNA structure reveals conservation in SARS-like coronaviruses. bioRxiv, 2020. 10.1101/2020.06.15.153197.

[ref41] Sun L.; Li P.; Ju X.; Rao J.; Huang W.; Ren L.; Zhang S.; Xiong T.; Xu K.; Zhou X.; et al. In vivo structural characterization of the SARS-CoV-2 RNA genome identifies host proteins vulnerable to repurposed drugs. Cell 2021, 184, 1865–1883.e20. 10.1016/j.cell.2021.02.008. 33636127 PMC7871767

[ref42] Pasquali S.; Gan H. H.; Schlick T. Modular RNA architecture revealed by computational analysis of existing pseudoknots and ribosomal RNAs. Nucleic Acids Res. 2005, 33, 1384–1398. 10.1093/nar/gki267. 15745998 PMC552955

[ref43] Gevertz J.; Gan H. H.; Schlick T. In vitro RNA random pools are not structurally diverse: A computational analysis. RNA 2005, 11, 853–863. 10.1261/rna.7271405. 15923372 PMC1370770

[ref44] Schlick T.; Yan S. Comprehensive Computational Chemistry, 1st ed.; Yáñez M.; Boyd R. J. Eds.; Elsevier, 2023, Vol. 3, pp. 886–894.

[ref45] Sato K.; Kato Y.; Hamada M.; Akutsu T.; Asai K. IPknot: Fast and accurate prediction of RNA secondary structures with pseudoknots using integer programming. Bioinformatics 2011, 27, i85–i93. 10.1093/bioinformatics/btr215. 21685106 PMC3117384

[ref46] Jain S.; Bayrak C. S.; Petingi L.; Schlick T. Dual Graph Partitioning Highlights a Small Group of Pseudoknot-Containing RNA Submotifs. Genes 2018, 9, 371 10.3390/genes9080371. 30044451 PMC6115904

[ref47] Zhu Q.; Petingi L.; Schlick T. RNA-As-Graphs Motif Atlas—Dual Graph Library of RNA Modules and Viral Frameshifting-Element Applications. Int. J. Mol. Sci. 2022, 23, 9249 10.3390/ijms23169249. 36012512 PMC9408923

[ref48] Dirks R.; Pierce N. A partition function algorithm for nucleic acid secondary structure including pseudoknots. J. Comput. Chem. 2003, 24, 1664–1677. 10.1002/jcc.10296. 12926009

[ref49] Wilkinson K. A.; Merino E. J.; Weeks K. M. Selective 2’-hydroxyl acylation analyzed by primer extension (SHAPE): Quantitative RNA structure analysis at single nucleotide resolution. Nat. Protoc. 2006, 1, 1610–1616. 10.1038/nprot.2006.249. 17406453

[ref50] Smola M. J.; Rice G. M.; Busan S.; Siegfried N. A.; Weeks K. M. Selective 2’-hydroxyl acylation analyzed by primer extension and mutational profiling (SHAPE-MaP) for direct, versatile and accurate RNA structure analysis. Nat. Protoc. 2015, 10, 1643–1669. 10.1038/nprot.2015.103. 26426499 PMC4900152

[ref51] Busan S.; Weeks K. M. Accurate detection of chemical modifications in RNA by mutational profiling (MaP) with ShapeMapper 2. RNA 2018, 24, 143–148. 10.1261/rna.061945.117. 29114018 PMC5769742

[ref52] Tomezsko P. J.; Corbin V. D. A.; Gupta P.; Swaminathan H.; Glasgow M.; Persad S.; Edwards M. D.; Mcintosh L.; Papenfuß A. T.; Emery A.; Swanstrom R.; Zang T.; Lan T. C. T.; Bieniasz P.; Kuritzkes D. R.; Tsibris A.; Rouskin S. Determination of RNA structural diversity and its role in HIV-1 RNA splicing. Nature 2020, 582, 438–442. 10.1038/s41586-020-2253-5. 32555469 PMC7310298

[ref53] Hajdin C. E.; Bellaousov S.; Huggins W.; Leonard C. W.; Mathews D. H.; Weeks K. M. Accurate SHAPE-directed RNA secondary structure modeling, including pseudoknots. Proc. Natl. Acad. Sci. U.S.A. 2013, 110, 5498–5503. 10.1073/pnas.1219988110. 23503844 PMC3619282

[ref54] Morandi E.; Manfredonia I.; Simon L. M.; Anselmi F.; van Hemert M. J.; Oliviero S.; Incarnato D. Genome-scale deconvolution of RNA structure ensembles. Nat. Methods 2021, 18, 249–252. 10.1038/s41592-021-01075-w. 33619392

[ref55] Zhang J.; Kobert K.; Flouri T.; Stamatakis A. PEAR: A fast and accurate Illumina Paired-End reAd mergeR. Bioinformatics 2014, 30, 614–620. 10.1093/bioinformatics/btt593. 24142950 PMC3933873

[ref56] Incarnato D.; Morandi E.; Simon L. M.; Oliviero S. RNA Framework: An all-in-one toolkit for the analysis of RNA structures and post-transcriptional modifications. Nucleic Acids Res. 2018, 46, e97 10.1093/nar/gky486. 29893890 PMC6144828

[ref57] Zubradt M.; Gupta P.; Persad S.; Lambowitz A. M.; Weißman J. S.; Rouskin S. DMS-MaPseq for genome-wide or targeted RNA structure probing in vivo. Nat. Methods 2017, 14, 75–82. 10.1038/nmeth.4057. 27819661 PMC5508988

[ref58] Yan S.; Schlick T. Heterogeneous and multiple conformational transition pathways between pseudoknots of the SARS-CoV-2 frameshift element. Proc. Natl. Acad. Sci. U.S.A. 2025, 122, e24179122. 10.1073/pnas.2417479122 PMC11789066 39854230

[ref59] Cao C.; Cai Z.; Xiao X.; Rao J.; Chen J.; Hu N.; Yang M.; Xing X.; Wang Y.; Li M.; et al. The architecture of the SARS-CoV-2 RNA genome inside virion. Nat. Commun. 2021, 12, 3917 10.1038/s41467-021-22785-x. 34168138 PMC8225788

[ref60] Huston N. C.; Wan H.; Strine M. S.; de Cesaris Araujo Tavares R.; Wilen C. B.; Pyle A. M. Comprehensive in vivo secondary structure of the SARS-CoV-2 genome reveals novel regulatory motifs and mechanisms. Mol. Cell 2021, 81, 584–598.e5. 10.1016/j.molcel.2020.12.041. 33444546 PMC7775661

[ref61] Johnson P. Z.; Simon A. E. RNAcanvas: Interactive drawing and exploration of nucleic acid structures. Nucleic Acids Res. 2023, 51, W501–W508. 10.1093/nar/gkad302. 37094080 PMC10320051

[ref62] Kelly J. A.; Olson A. N.; Neupane K.; Munshi S.; San Emeterio J.; Pollack L.; Woodside M. T.; Dinman J. D. Structural and functional conservation of the programmed −1 ribosomal frameshift signal of SARS coronavirus 2 (SARS-CoV-2). J. Biol. Chem. 2020, 295, 10741–10748. 10.1074/jbc.AC120.013449. 32571880 PMC7397099

[ref63] Hsu C.-F.; Chang K.-C.; Chen Y.-L.; Hsieh P.-S.; Lee A.-I.; Tu J.-Y.; Chen Y.-T.; Wen J.-D. Formation of frameshift-stimulating RNA pseudoknots is facilitated by remodeling of their folding intermediates. Nucleic Acids Res. 2021, 49, 6941–6957. 10.1093/nar/gkab512. 34161580 PMC8266650

