# Supplementary material for: A Cascade of Conformational Switches in SARS-CoV-2 Frameshifting: Coregulation by Upstream and Downstream Elements
Source: Biochemistry. 2025 Feb 5;64(4):953–66. doi: 10.1021/acs.biochem.4c00641 (PMC11840926; doi:10.1021/acs.biochem.4c00641)
Supplement: Supplementary file 1 — bi4c00641_si_001.pdf [file bi4c00641_si_001.pdf]

Supplementary Information for A cascade of conformational switches in SARS-CoV-2 frameshifting: co-regulation by upstream and downstream elements

Samuel Lee and Shuting Yan,[1,2] Abhishek Dey,[3] Alain Laederach,[4] and Tamar Schlick*[2,5,6,7,a)]

[1)] *Both contributed equally to this work as first co authors.*

[2)] *Department of Chemistry, New York University, New York, NY 10003 U.S.A.*

[3)] *Department of Biotechnology, National Institute of Pharmaceutical Education and Research-Raebareli (NIPER-R), Lucknow, 226002, Uttar Pradesh, India*

[4)] *Department of Biology, University of North Carolina at Chapel Hill, Chapel Hill, NC 27599, U.S.A.*

[5)] *Courant Institute of Mathematical Sciences, New York University, New York, NY 10012 U.S.A.*

[6)] *NYU-ECNU Center for Computational Chemistry, NYU Shanghai, Shanghai 200062, P.R. China*

[7)] *NYU Simons Center for Computational Physical Chemistry, New York University, New York, NY 10003 U.S.A*

(Dated: 13 January 2025)

[a)] Electronic mail: schlick@nyu.edu

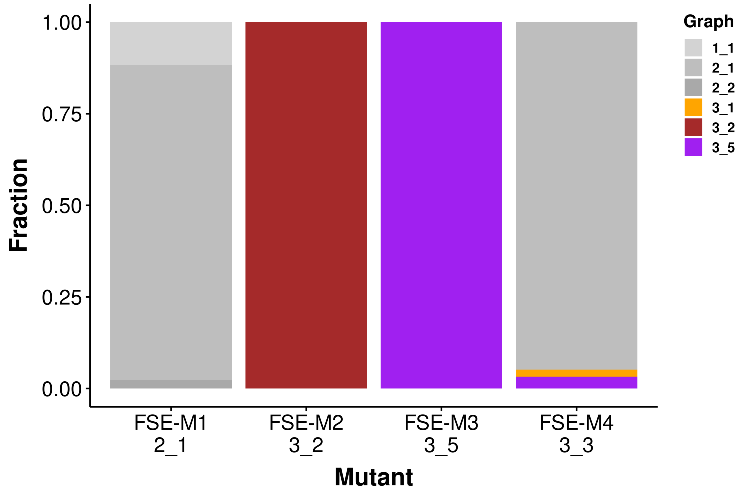

FIG. S1. Folding of 77-nt double mutants in our previous work[1] embedded in 114-nt and examined by DMS-MaP in Pekarek et al.[2]. The fractions of RNA folds are calculated from DREEM clustering (K=3) and ShapeKnots predictions weighted by Boltzmann factor. The topology of 77-nt FSE regions are represented via dual graph IDs. The target fold of each mutant is labeled under it name. Note that FSE-M1, M2 and M3 embedded in long constructs predict folds as designed for 77-nt, but FSE-M4 yield mostly 2_1 because the S3 loop that should be pseudoknotted with the downstream stem is flexible/unstable according to the DMS data.

I. COMPARISON BETWEEN OUR LANDSCAPE PREDICTIONS AND PUBLISHED DMS CHEMICAL PROBING EXPERIMENTS

We compare our predicted structures with DMS-MaP results by Pekarek et al.[2] for the most comparable sequence/construct. Our 143-nt construct with 34 residues upstream of the slippery site and 25 residues downstream of the 77-nt central FSE aligns well with the 141-nt FSE-V2 construct[3] (only 2-nt difference at the 5′-end). In Figure S4A, we show consistent dominant 3_6 FSE predicted in our landscape in agreement with the findings of Pekarek et al.[2]. The differences include the minor 3_3 conformation predicted in our landscape (21.67% at 143-nt) and the small hairpin following AH in the 3_6-containing motifs (see complete prediction profile in Figure S2). The dominant 3_6-containing motif for the AH-FSE region predicted in our landscape is conserved from shorter sequences, such as 118-nt, to sequences that match with experiments in[2].

II. 156-NT DMS DATA AND COMPUTATIONAL LANDSCAPES ASSESSMENT

To assess our computational predictions, we also perform DMS chemical probing experiments on 156-nt constructs (see Methods) and analyze the information using DREEM[4] and DRACO[5] (see Figure S4B and Figure S3). Sequencing reads are clustered and the reactivity profiles guide RNA predictions via ShapeKnots[6]. For the 156-nt DMS data analyzed here, dominant folds contain a long AS1, AH and 2_2 FSE, with S1 absent but S2 and S3 of 3_6 present. S1 cannot form because of the extra base pairs (AU and GC pairs) in AS1 (in Figure S4B). DREEM also predicts an alternative 2_1 FSE, where FSE S1 and S3 persist without S2. In comparison, our computed landscapes for this length in Figure 2B predict a minority of 2_2 (gray, 3.35%), 49.11% AH-containing 3_6 motif (8-bp AS1), and 33.22% 3_8 containing motif with shifted S1 and 12-bp AS1.

All these motifs are consistent with the mechanistic picture emerging from our landscape (next section) and the fact that DREEM and DRACO programs do not favor formation of pseudoknots at this long sequence length.

In our landscape, as sequence length increases from 141 to 156-nt, the distribution of different motifs (mainly 3_6 FSE in combination with an 8-bp AS1 and 3_8 FSE in combination with a 12-bp AS1) changes consistently with the increasing fraction of motifs with a 12-bp AS1. In these motifs, S1 is disrupted, consistent with DMS data, confirming that S1 can form only when AS1 is shorter or equal to 10-bp, though the 3_8 pseudoknot with a shifted short stem may also emerge.

The reliability of our NUPACK landscapes can be determined by examining the predictions on 77-nt wildtype and motif strengthening mutants (in Table S2). For long sequences, such as 156-nt, 3_6 FSE is also predicted by other softwares (Figure S4) despite different base pairs variations for the AS1 region. The DMS-guided predictions via DREEM and DRACO also exhibit similar behavior. DREEM and DRACO agree with NUPACK at short sequences, such as 77-nt, but inconsistencies quickly arise for longer RNAs, such as 87-nt and 114-nt, as we show in Table S3. Our close examination make clear that caution is warranted when interpreting the results for both pure computational and chemical probing

guided predictions at long sequences, especially regarding pseudoknots, which tend to be underpredicted by softwares such as DREEM, DRACO and DANCE-MaP[7].

III. M3_5 SARS-COV-2 CONFORMATIONAL LANDSCAPE

The conformational landscapes reveal that the M3_5 mutant refolds as downstream residues are added (Figure 6)). Many of the resulting motifs hold a 3_3 FSE instead of the expected 3_5 FSE, underscoring the minor role of the three-way junction fold in the conformational ensemble. A possible AH and 3_5-containing motif is 6_52 at 140$\sim$144-nt for 5-10% shown in Figure S7. The formation of 3_3 S2 in 6_458 (Figure S6C) is favored over that of 3_5 S2 in 6_52 for most of the motifs.

Similar to the wildtype variant, many of these motifs possess AS1 after adding upstream residues, preventing 3_5 S2 from forming. A motif with AS1 intertwined with a shifted 3_5 S2 exists at 122$\sim$126-nt very rarely for 2-6% (Figure S1).

IV. M3_5 SARS-COV-2 RAG-IF MUTATION DESIGN FOR 3_5 AT LONG SEQUENCES

To construct a more stable 3_5 FSE for long sequences, we utilize our mutation design algorithm, RAG-IF (see **Methods**), to search for minimal mutations.

We focus on the downstream residues of 3_3 S2, hoping to simultaneously break 3_3 S2 and block 3_6 S2 to make 3_5 S2 favorable. We ran RAG-IF for each motif that did not have a 3_5 FSE, hoping to find mutations that would cause a motif to maintain AH and a stable downstream 3_5 FSE. After mutating all eight motifs, we looked for commonalities among the many possible mutation sequences for each motif.

Ultimately, we found seven total mutations: [G16U, U17G, G21A, C23U, C24A, C47A, G52U] (Figure S9). Specifically, C23U and C24A break two G-C pairs in 3_3 S2, weakening 3_3 S2 and making the formation of 3_5 S2 much more favorable. The three mutations G21A, C23U, and C24A strengthen 3_5 S1 and block the formation of 3_6 S2. Together, these mutations break 3_3 S2, block the formation of 3_6 S2, and strengthen 3_5 FSE.

TABLE S1. Full sequences of the mutants in this work. Mutations are colored and in bold. The wildtype, M3_3, M3_5, M3_6 and M3_5$^+$ are 118-nt; M3_6$^+$ is 132-nt; and M2_2 is 156-nt.

System	Sequence
Wildtype	AACCCAUGCUUCAGUCAGCUGAUGCACAAUCGUUUUUAAAC<br>GGGUUUGCGGUGUAAGUGCAGCCCGUCUUACACCGUGCGGCACAGGCACUAGUACUGAUGUCGUAUACAGGGCUUUU
M3_3	AACCCAUGCUUCAGUCAGCUGAUGCACAAUCGUUUUUAAAC<br>GGG**C**UUGCGGUGUAAGUGCAGCCCGUCUUACACCGUGCGGCACAGGCACUAGUACUGAUGUCGUAUACAG**AU**CUUUU
M3_5	AACCCAUGCUUCAGUCAGCUGAUGCACAAUCGUUUUUAAAC<br>GGGUUUGCGGUGUAAGUGCAGCCCGUCUUACACCGUGCGGCACAGGCACUAGUACUGAUGUCGUAUACAGG**C**C**C**UUU
M3_6	AACCCAUGCUUCAGUCAGCUGAUGCACAAUCGUUUUUAAAC<br>GG**UA**UUGCGGUGUAAGU**AA**AGCCCGUCUUACACCGUGCGGCACAGGCACUAGUACUGAUGUCGUAUA**AC**GGGCUUUU
M3_5$^+$	AACCCAUGCUUCAGUCAGCUGAUGCACAAUCGUUUUUAAAC<br>GGGUUUGCGGUGUAA**UG**GCA**A**C**UA**GUCUUACACCGUGCGGCACAGG**A**ACUA**U**UACUGAUGUCGUAUACAGG**C**C**C**UUU
M3_6$^+$	UGAUCAACUCCGCG<br>AACCCAUGCUUCAGUCAGCUGAUGCACAAUCGUUUUUAAAC<br>**UUU**UUUGCGGUGUAAGUGCAGCCCGUCUUACACCGUGCGGCACAGGCACUAGUACUGAUGUCGUAUACAGGGCUUUU
M2_2	ACUCCGCGAACCCAUGCUUCAGUCAGCUGAUGCACAAUCGUUUUUAAAC<br>GGGUUUGCGGUGUA**UA**UGCAGCCCGUCUU**U**CACCGUGCGGCACAGGCACUAGUACUGAUGUCGUAUACAGGGCUUUU<br>GACAUCUACAAUGAUAAAGUAGCUGGUUUU

TABLE S2. Secondary structures predicted by eight software packages for SARS-CoV-2 77-nt FSE and motif strengthening mutants. MFE structures are presented in dual graph notations.

Software	77WT	M3_6	M3_5	M3_3
**ProbKnot**	3_5	✓3_6	✓3_3	✓3_5
**Pknots**	✓3_6	✓3_6	✓3_3	✓3_5
**Ipknot**	2_1	✓3_6	No graph match	✓3_5
**vsfold5**	4_7 (3_3-containing)	✓3_6	4_3 (3_3-containing)	4_3 (3_3-containing)
**pKiss**	✓3_6	✓3_6	✓3_3	✓3_5
**Vfold2D**	✓3_6	3_3	✓3_3	✓3_5
**Knotty**	4_7 (3_3-containing)	✓3_6	4_7 (3_3-containing)	✓3_5
**NUPACK**	✓3_6	✓3_6	✓3_3	✓3_5
**ShapeKnots**	3_5	✓3_6	2_1	✓3_5

When examining the motifs created from this new mutation sequence (M3_5$^+$), the seven mutations clearly help stabilize 3_5 FSE over a wide range of lengths (Figure S9).

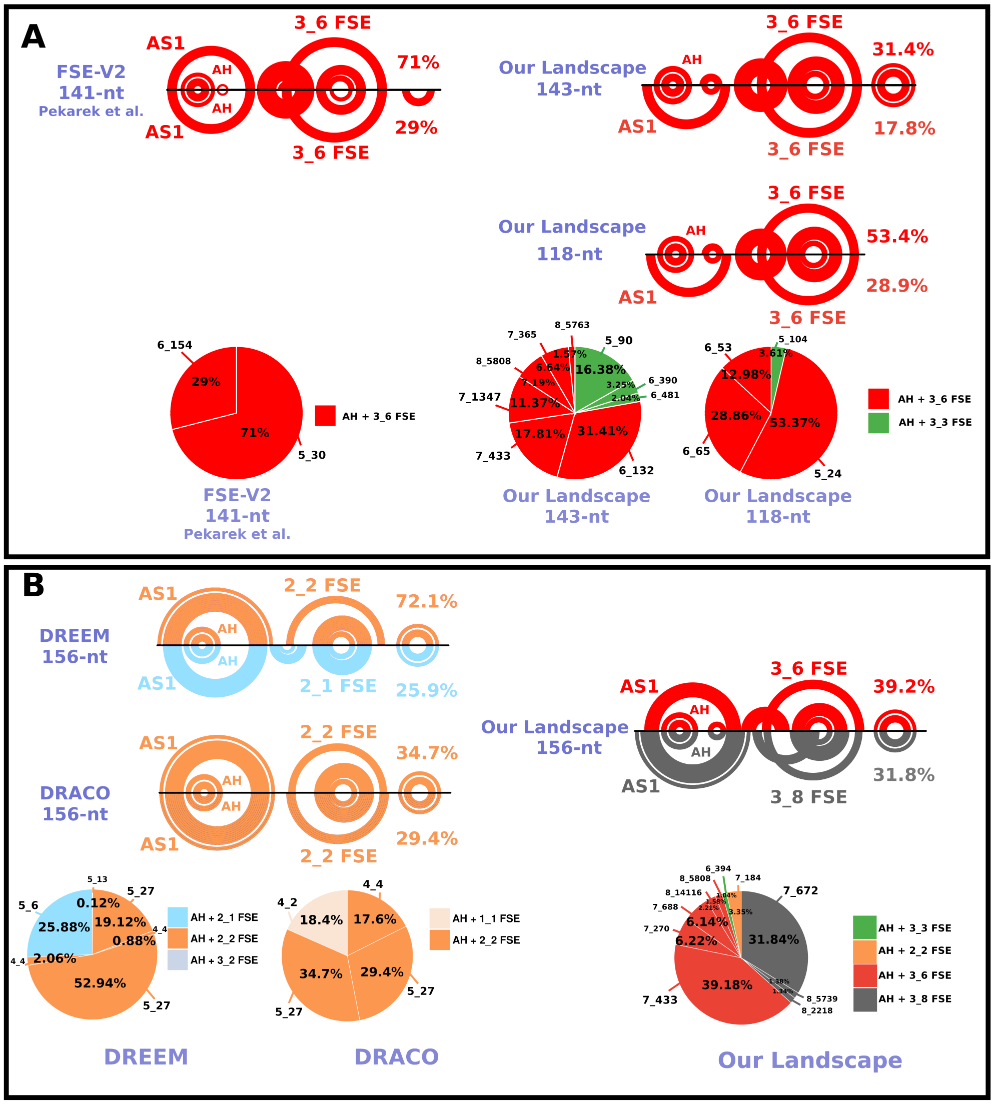

FIG. S2. Comparison of our landscape predictions with DMS-MaP chemical probing supported structures. Two folds for each sequence and prediction combination with the largest fraction are shown in arc plots. (A) Comparison of 143-nt from wildtype downstream landscape and 141-nt FSE-V2 construct by Pekarek et al.[2]. (B) Comparison of 156-nt from the wildtype long sequence landscape and the same construct from DMS-MaPseq chemical probing experiment followed by DREEM[4] and DRACO[5] clustering and ShapeKnots[6] predictions. The overall distributions of motifs for DREEM, DRACO and our landscapes are shown in a pie chart format, and full details of the graph motifs are given in Figure S3.

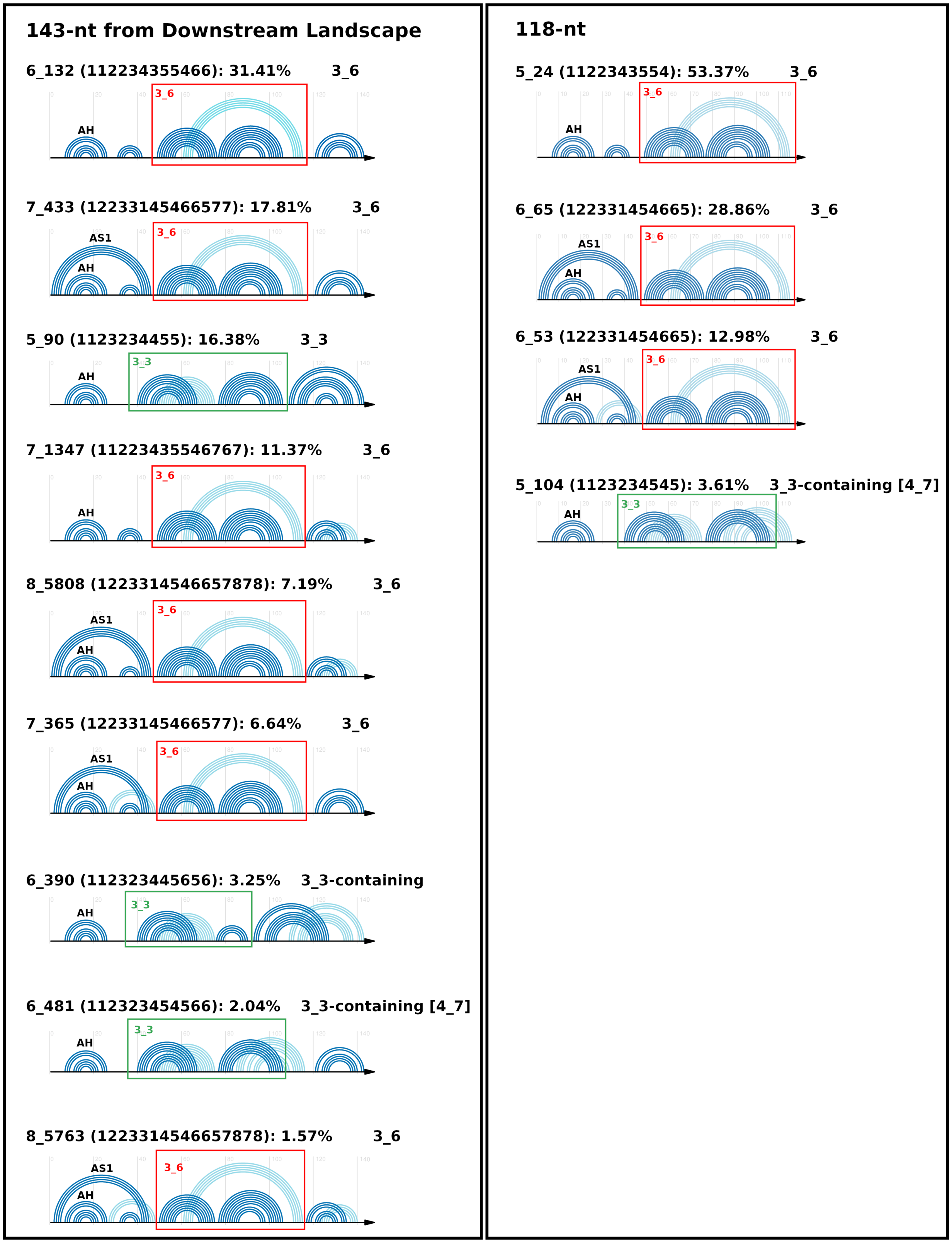

FIG. S3. Secondary structures of 143-nt and 118-nt FSE from the downstream landscape. Corresponding fractions are labeled for each motif. The dominant motifs are AH + 3_6 for 95.92% at 118-nt and for 75.99% at 143-nt. The minor motifs are AH + 3_3 for 3.61% at 118-nt and for 21.67% at 143-nt.

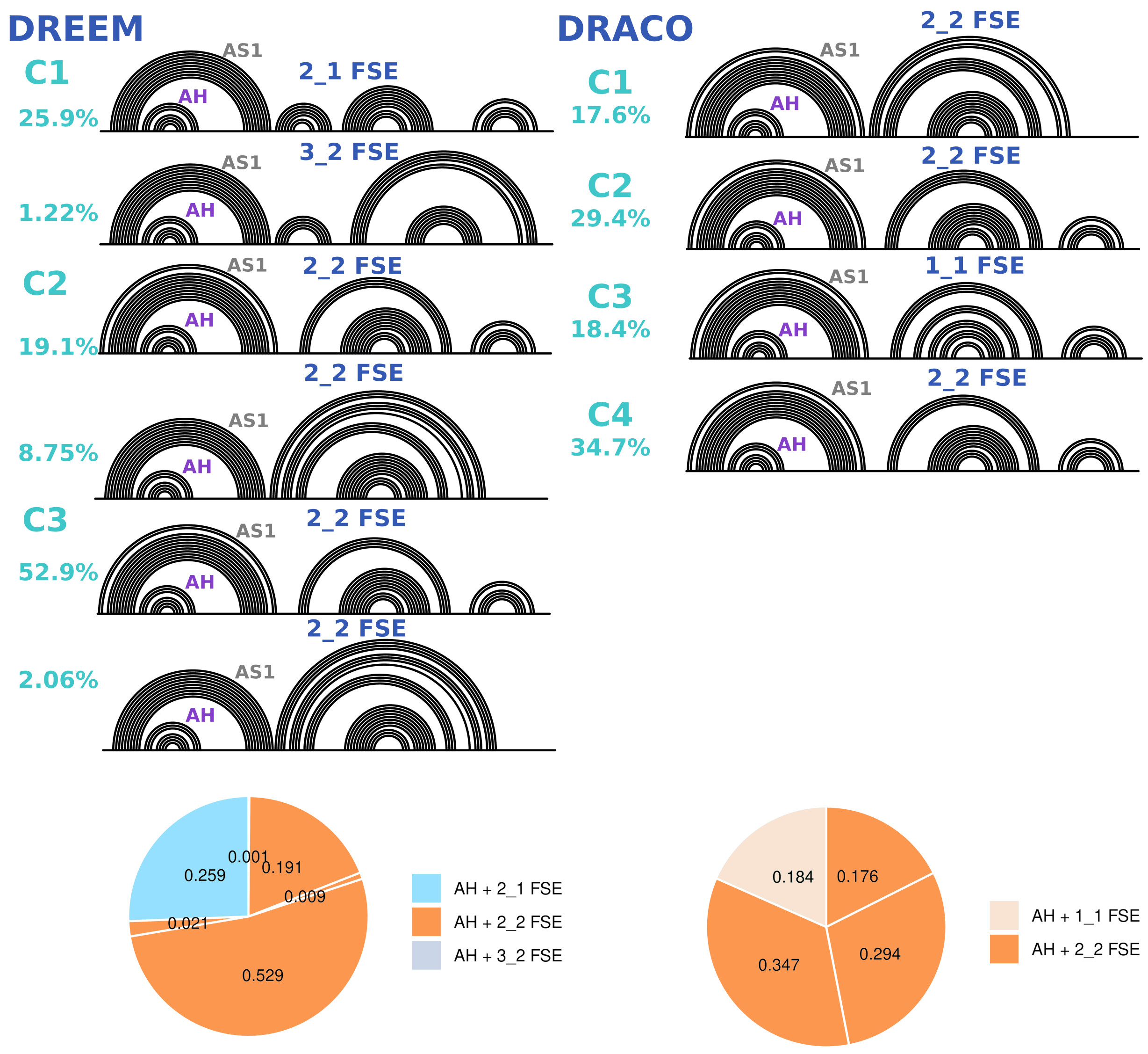

FIG. S4. The 156-nt construct is investigated by DMS-MaPseq and the folds are predicted by ShapeKnots[6] guided by DMS reactivity profiles. DREEM[4] and DRACO[5] have been applied to obtain clusters of alternative folds and produce DMS reactivity profile. DREEM were run with the setting K(number of clusters)=3, and DRACO produces 4 clusters.

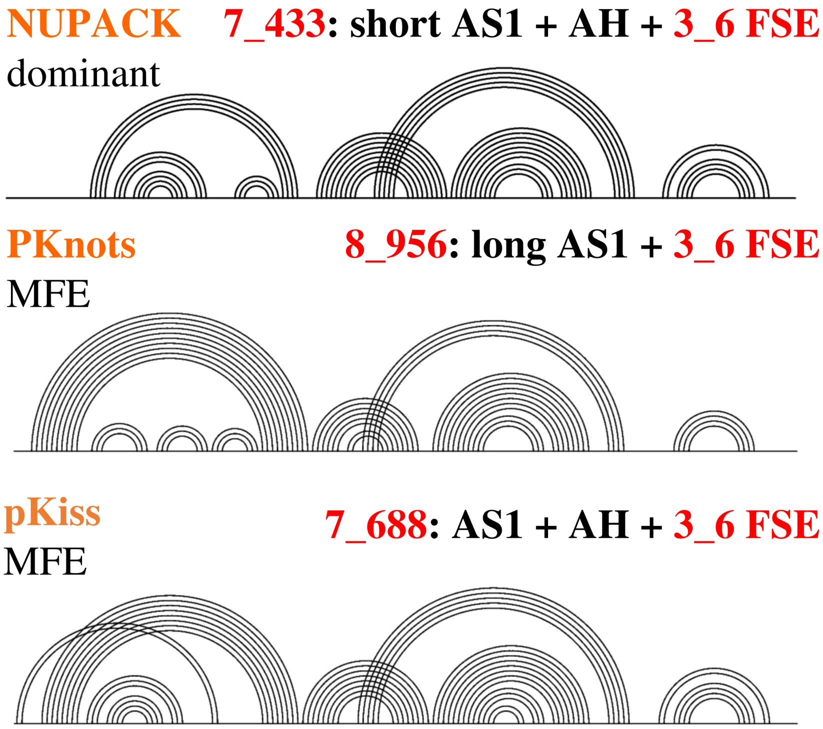

FIG. S5. Predictions of 156-nt FSE construct by selected software packages (NUPACK[8], PKNOTS[9] and pKiss[10]).

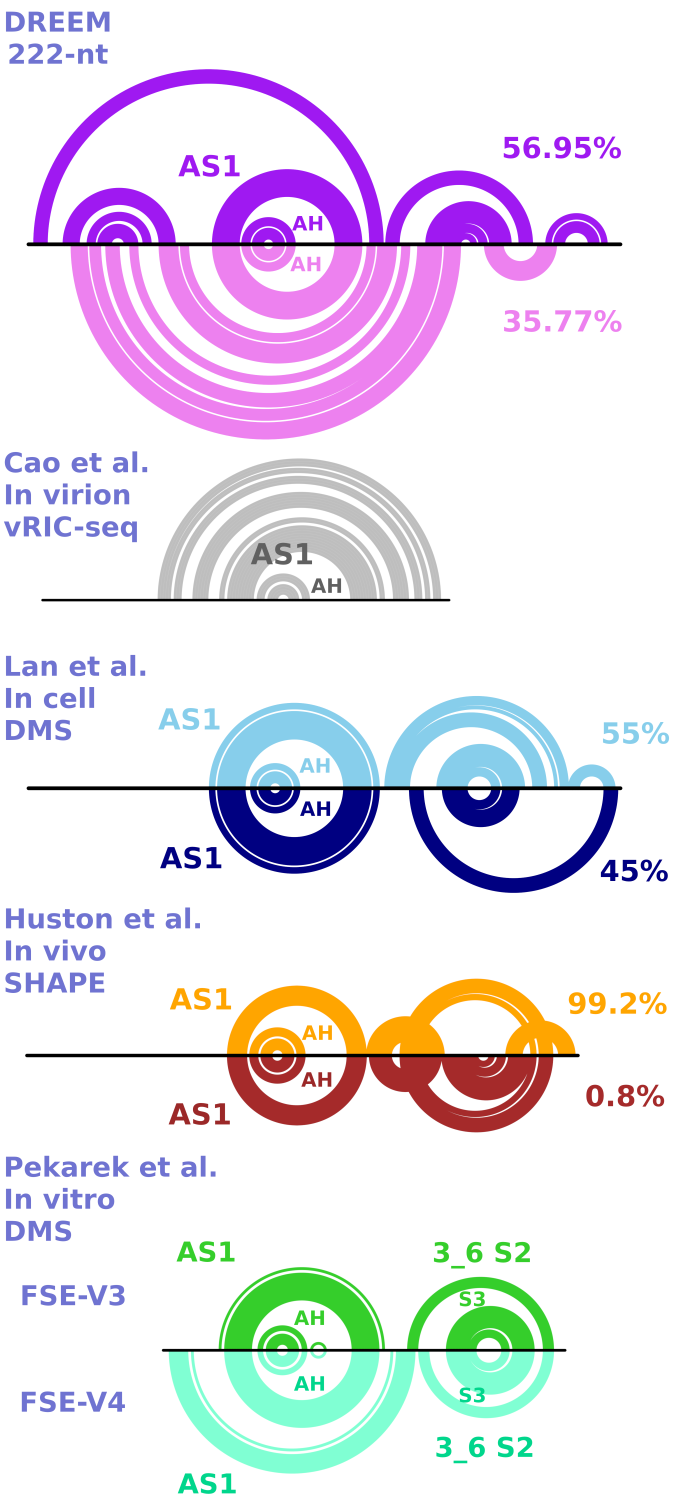

FIG. S6. 222-nt FSE-containing construct characterized by DMS and structures characterized in different experimental conditions in the literature[11–13].

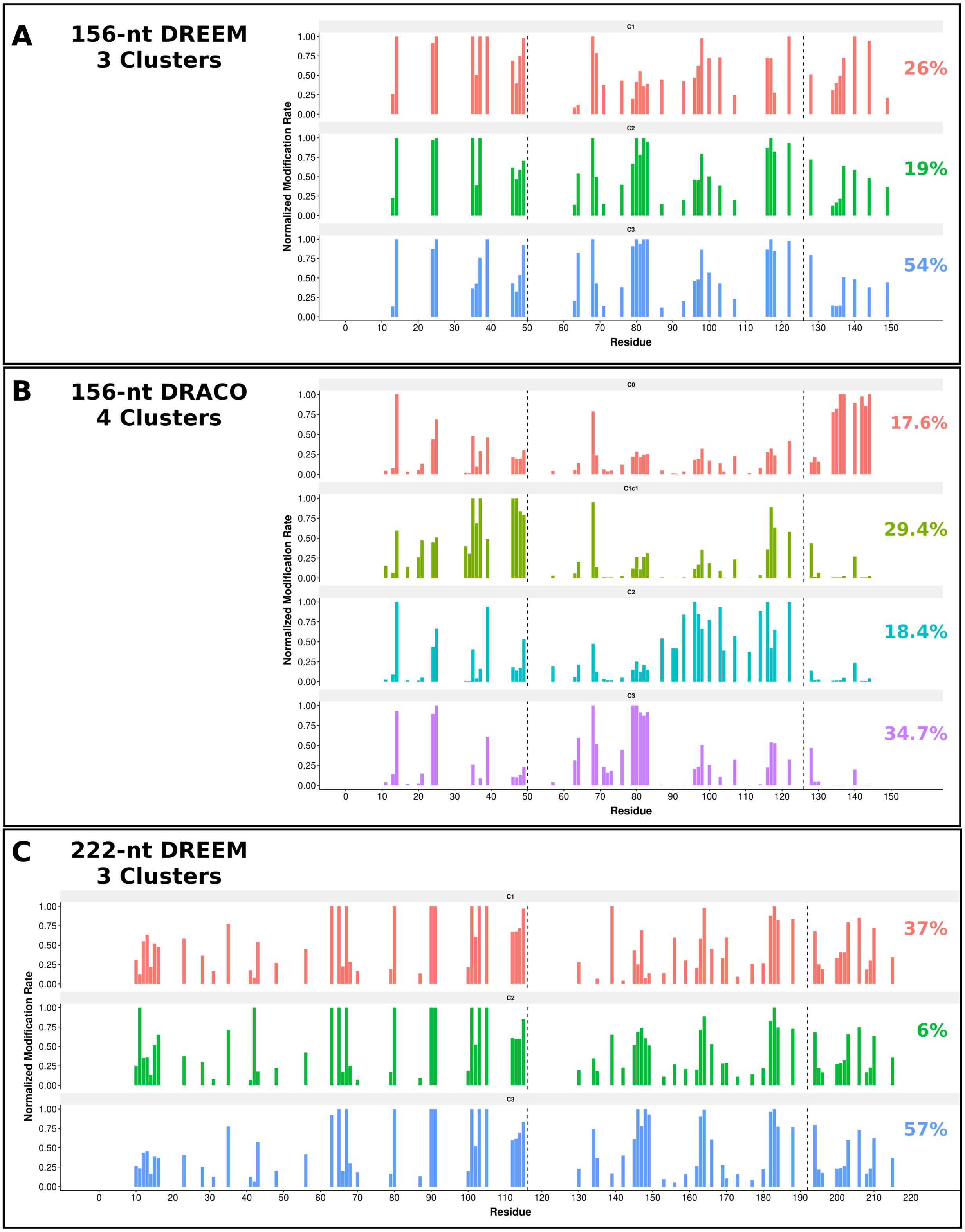

FIG. S7. DMS-MaPseq reactivity data of 156-nt and 222-nt FSE-containing sequences processed by DREEM (A: 156-nt, C: 222-nt) and DRACO (B: 156-nt). Dashed lines indicate the central 77-nt FSE. The fraction of each cluster is labeled.

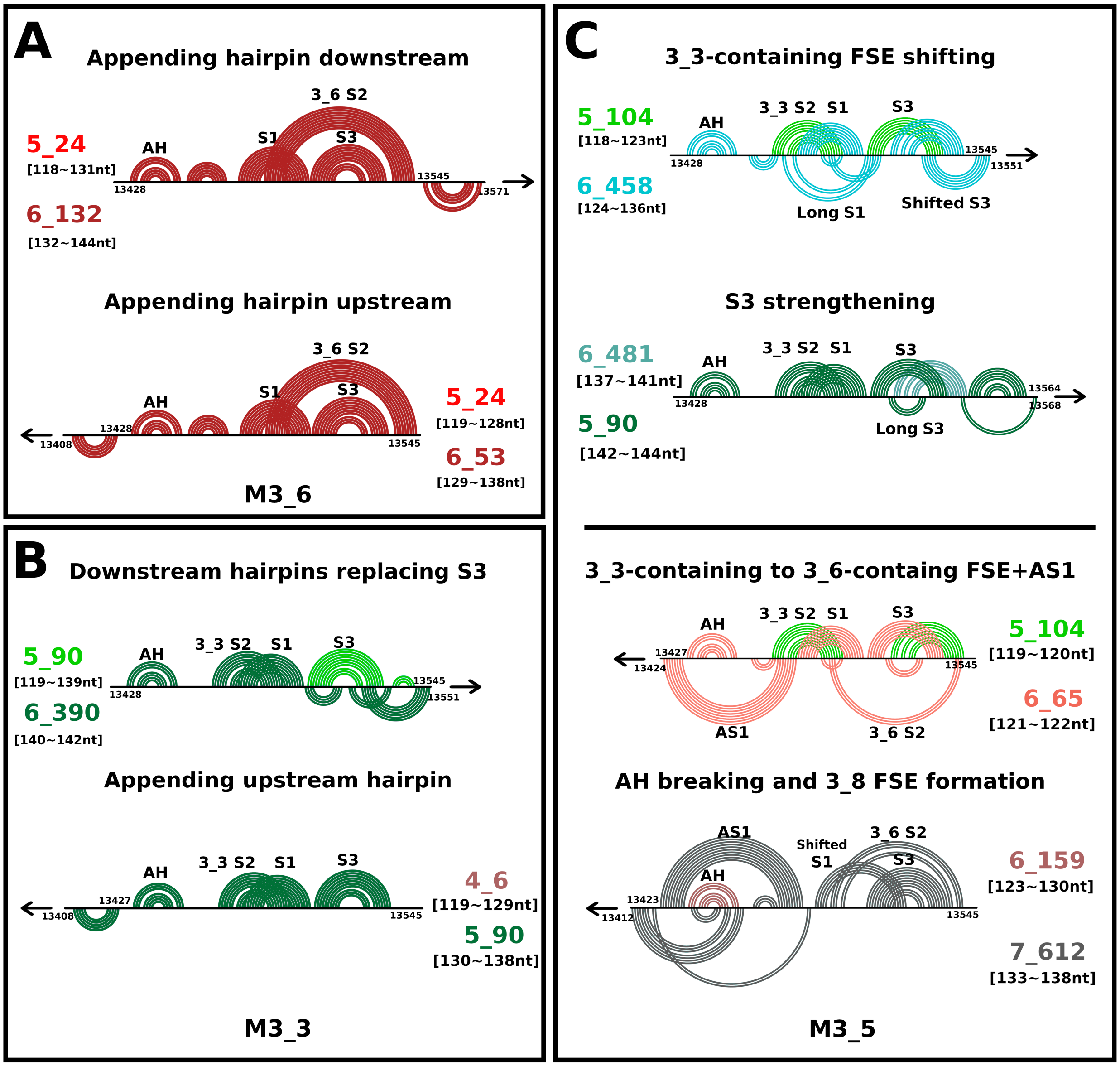

FIG. S8. Transitions of 2D folding from the dominant motifs of FSE mutant systems in this work with additional downstream and upstream sequence are shown in arc plots, for (A-C) the motif strengthening mutants M3_6 (A), M3_3 (B), and M3_5 (C). The 2D arc plots for two motifs in different colors are overlapped to show the differences in RNA folding. 3_6 FSE containing motifs are colored in red; similarly, 3_3 FSE containing motifs are in green, and 3_8-containing motifs are in gray. For the M3_6 (A) and M3_3 (B), two transitions are displayed, top for downstream expansion and bottom for upstream expansion (expansion indicated by the arrows). For M3_5 (C), four transitions are displayed, with the top two transitions indicating downstream sequence expansion and the bottom two indicating upstream sequence expansion. The overlapped arc plots depict each transition, with added pairs in the second motif at the bottom and common stems at the top.

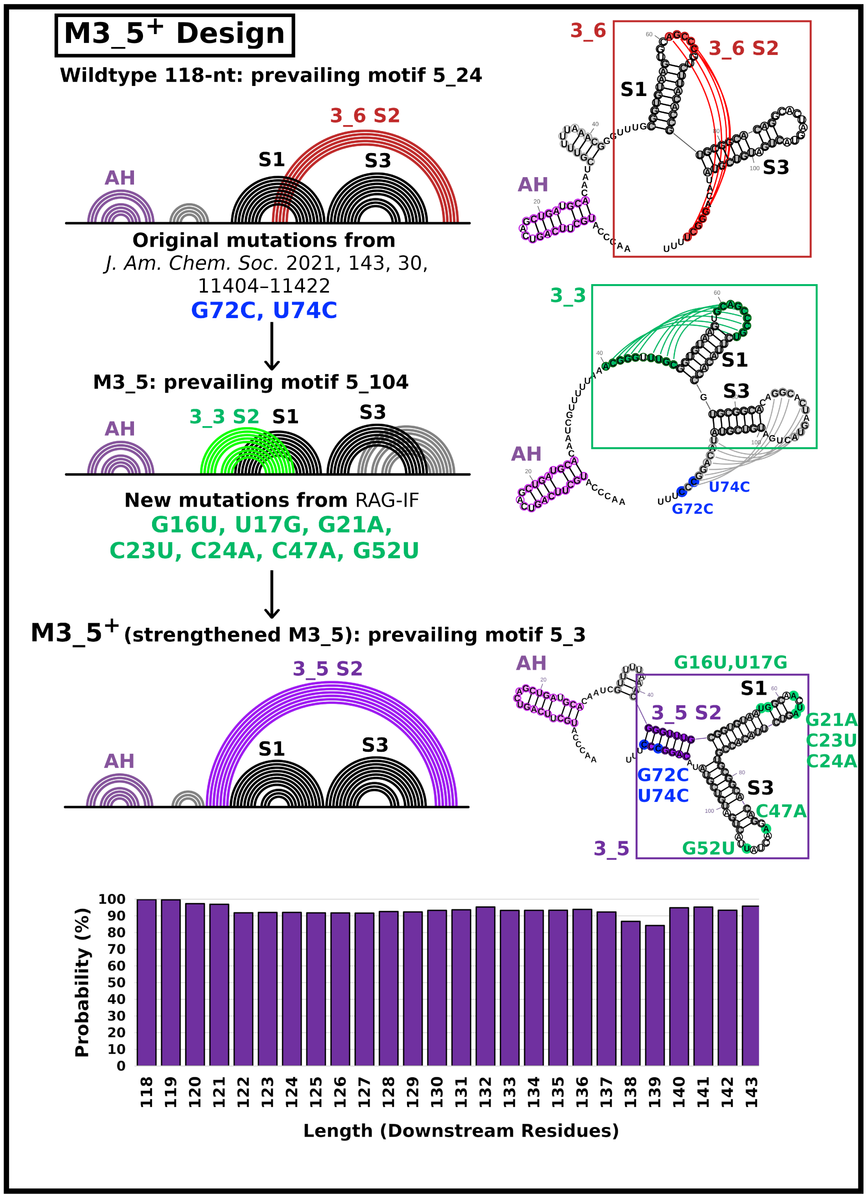

FIG. S9. Mutant designs to stabilize 3_5 (M3_5$^+$) in long FSE-containing sequences. To design the mutant (M3_5$^+$) that supports 3_5 junction formation, we use RAG-IF and multiple 2D structure prediction program screening to determine the minimal mutations. The original mutations in M3_5[14] are denoted in blue. To stabilize 3_5 for long sequence contexts, we identify 7 mutations by RAG-IF and add to M3_5. New mutations from this work are denoted in green. The conformational landscapes are calculated for each mutant to examine motif stability under a long sequence context.

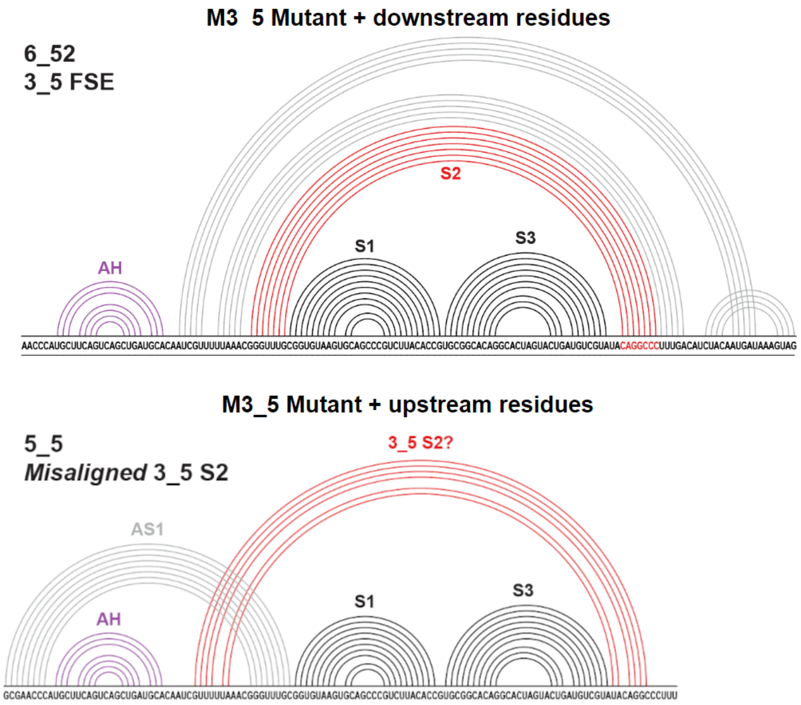

FIG. S10. Motifs containing AH and 3_5 FSE that rarely appear for M3_5 system.

TABLE S3. Boltzmann fractions computed for 77-nt, 87-nt and 114-nt SARS-CoV-2 FSE constructs using DREEM and DRACO.

System	Motif	DREEM	DRACO
Wildtype 77-nt	3_6	0.935	0.657
	3_5	0.0006	0.002
	3_3	0.057	0.145
	2_1	0	0.195
Wildtype 87-nt	3_6	0.89	0.16
	3_5	0.03	0.13
	3_3	0.06	0.57
	2_1	0	0.14
Wildtype 114-nt (Pekarek et al. FSE-V1)	3_3	0.95	NA
	3_5	0.03	NA
	2_1	0.01	NA

REFERENCES

[1]T. Schlick, Q. Zhu, S. Jain, and S. Yan, "Structure-altering mutations of the SARS-CoV-2 frameshifting RNA element," Biophys. J. **120**, 1040–1053 (2021).

[2]L. Pekarek, M. M. Zimmer, A.-S. Gribling-Burrer, S. Buck, R. Smyth, and N. Caliskan, "Cis-mediated interactions of the SARS-CoV-2 frameshift RNA alter its conformations and affect function," Nucleic Acids Res. **51**, 728–743 (2023).

[3]W. Sanders, E. J. Fritch, E. A. Madden, R. L. Graham, H. A. Vincent, M. T. Heise, R. S. Baric, and N. J. Moorman, "Comparative analysis of coronavirus genomic RNA structure reveals conservation in SARS-like coronaviruses," bioRxiv , 2020.06.15.153197 (2020).

[4]P. J. Tomezsko, V. D. A. Corbin, P. Gupta, H. Swaminathan, M. Glasgow, S. Persad, M. D. Edwards, L. Mcintosh, A. T. Papenfuss, A. Emery, R. Swanstrom, T. Zang, T. C. T. Lan, P. Bieniasz, D. R. Kuritzkes, A. Tsibris, and S. Rouskin, "Determination of RNA structural diversity and its role in HIV-1 RNA splicing," Nature **582**, 438–442 (2020).

[5]E. Morandi, I. Manfredonia, L. M. Simon, F. Anselmi, M. J. van Hemert, S. Oliviero, and D. Incarnato, "Genome-scale deconvolution of RNA structure ensembles," Nat. Methods **18**, 249–252 (2021).

[6]C. E. Hajdin, S. Bellaousov, W. Huggins, C. W. Leonard, D. H. Mathews, and K. M. Weeks, "Accurate SHAPE-directed RNA secondary structure modeling, including pseudoknots," Proc. Natl. Acad. Sci. U.S.A. **110**, 5498–5503 (2013).

[7]A. Dey, S. Yan, T. Schlick, and A. Laederach, "Abolished frameshifting for predicted structure-stabilizing SARS-CoV-2 mutants: Implications to alternative conformations and their statistical structural analyses," (2024), RNA, In Press.

[8]J. N. Zadeh, C. D. Steenberg, J. S. Bois, B. R. Wolfe, M. B. Pierce, A. R. Khan, R. M. Dirks, and N. A. Pierce, "NUPACK: Analysis and design of nucleic acid systems," J. Comput. Chem. **32**, 170–173 (2011).

[9]E. Rivas and S. R. Eddy, "A dynamic programming algorithm for RNA structure prediction including pseudoknots11Edited by I. Tinoco," J. Mol. Biol. **285**, 2053–2068 (1999).

[10]S. Janssen and R. Giegerich, "The RNA shapes studio," Bioinformatics **31**, 423–425 (2015).

[11]T. C. T. Lan, M. F. Allan, L. E. Malsick, J. Z. Woo, C. Zhu, F. Zhang, S. Khandwala, S. S. Y. Nyeo, Y. Sun, J. U. Guo, M. Bathe, A. Näär, A. Griffiths, and S. Rouskin, "Secondary structural ensembles of the SARS-CoV-2 RNA genome in infected cells," Nat.

Commun. **13**, 1128 (2022).

[12]N. C. Huston, H. Wan, M. S. Strine, R. de Cesaris Araujo Tavares, C. B. Wilen, and A. M. Pyle, "Comprehensive in vivo secondary structure of the SARS-CoV-2 genome reveals novel regulatory motifs and mechanisms," Mol. Cell **81**, 584–598.e5 (2021).

[13]C. Cao, Z. Cai, X. Xiao, J. Rao, J. Chen, N. Hu, M. Yang, X. Xing, Y. Wang, M. Li, B. Zhou, X. Wang, J. Wang, and Y. Xue, "The architecture of the SARS-CoV-2 RNA genome inside virion," Nat. Commun. **12**, 3917 (2021).

[14]T. Schlick, Q. Zhu, A. Dey, S. Jain, S. Yan, and A. Laederach, "To Knot or Not to Knot: Multiple Conformations of the SARS-CoV-2 Frameshifting RNA Element," J. Amer. Chem. Soc. **143**, 11404–11422 (2021).
